# Preventing late corrections reveals anticipatory motor control

**DOI:** 10.3389/fnsys.2026.1892135

**Published:** 2026-08-28

**Authors:** Inmaculada Márquez, Luis Lemus, Mario Treviño

**Affiliations:** 1 Departamento de Psicología, Centro Universitario de la Ciénega, Universidad de Guadalajara, Ocotlán, Mexico; 2 Laboratorio de Neurofisiología, Departamento de Bioingeniería Traslacional, Centro Universitario de Ciencias Exactas e Ingenierías, Guadalajara, Jalisco, Mexico; 3 Laboratorio de Plasticidad Cortical y Aprendizaje Perceptual, Instituto de Neurociencias, Universidad de Guadalajara, Guadalajara, Jalisco, Mexico; 4 Departamento de Neurociencia Cognitiva, Instituto de Fisiología Celular, Universidad Nacional Autónoma de México, Mexico City, Mexico

**Keywords:** gaze-hand coordination, predictive visuomotor control, preemptive motor planning, sensorimotor prediction, target interception

## Abstract

Successful interception requires combining predictive planning with continuous sensory feedback, yet these processes are difficult to dissociate because they operate simultaneously throughout movement. Here, we asked how predictive control is expressed when late online corrections are made ineffective but visual information is left intact. Participants performed a visuomotor interception task in which they moved a horizontal cursor to intercept a falling target at its predicted landing position. On selected trials, the cursor became temporarily unresponsive immediately before interception, preventing late motor corrections while leaving the target continuously visible. Manual and gaze behavior were quantified using two complementary root-mean-square error (RMSE) measures that tracked alignment with either the target’s instantaneous position or its landing position. Under normal conditions, manual movements converged on the predicted landing position earlier on successful than on unsuccessful trials; trial-averaged gaze showed no comparable effect. Increasing cursor-freeze durations progressively reduced interception accuracy but shifted manual behavior toward earlier alignment with the landing position before the freeze began, indicating a redistribution of control from late feedback corrections toward earlier predictive planning. These findings show that when late online corrections become ineffective, humans compensate by committing earlier to predicted future states. This redistribution was large and consistent in manual behavior, and considerably harder to detect in trial-averaged gaze.

## Introduction

Successful interception requires acting ahead of sensorimotor delays. Because neural processing and biomechanical constraints prevent purely reactive behavior, the nervous system must continuously estimate future target states and generate motor commands before sensory errors arise (Kawato, 1999; Márquez and Treviño, 2026; Todorov and Jordan, 2002; Wolpert et al., 1995). Visuomotor interception provides an ideal framework for studying this process because successful performance depends on continuously integrating internal predictions with incoming sensory information. Rather than reflecting either predictive or reactive control alone, interception emerges from the dynamic interaction between anticipatory planning and online feedback throughout movement execution (Brenner and Smeets, 2011, 2018; Franklin and Wolpert, 2011; Márquez et al., 2024).

Prediction is expressed not only in whether a movement succeeds, but also in how it unfolds over time. During successful interception, movements progressively commit toward the future interception point while remaining sufficiently flexible to accommodate sensory feedback when necessary. Time-resolved analyses, therefore, provide a more direct window into predictive control than endpoint accuracy alone (Elliott et al., 2001; Márquez et al., 2024; Márquez and Treviño, 2024a; Mrotek and Soechting, 2007; Senot et al., 2008).

Gaze contributes to this process in multiple ways. During interception, gaze alternates among tracking the moving target, sampling future interception locations, and monitoring ongoing manual performance through coordinated smooth pursuit, predictive saccades, and anticipatory fixations (Fooken et al., 2021; Johansson et al., 2001; Land, 2006; Treviño and Márquez, 2025; Zhao et al., 2019). Because these functions vary across trials and even within individual movements, averaging gaze trajectories can obscure predictive behavior that is clearly expressed on single trials. Consequently, gaze provides valuable but indirect evidence of predictive control, making complementary measures of manual behavior essential.

Most previous studies have investigated predictive control by manipulating sensory information, for example, through target occlusion, delayed visual feedback, or altered sensory consequences of action (Dignath and Janczyk, 2017; Galea et al., 2011; Knelange and López-Moliner, 2020; Shadmehr and Mussa-Ivaldi, 1994; Weber and Bellebaum, 2024). These manipulations do increase reliance on internal predictions, but they also alter the perceptual evidence available to the observer and the motor commands that depend on that evidence. Consequently, behavioral adaptations cannot be unambiguously attributed to perceptual or motor processes.

Here, we addressed this limitation by manipulating a different stage of the sensorimotor loop. Rather than reducing visual information, we selectively eliminated the effectiveness of late online motor corrections while leaving visual information completely unchanged. Participants continuously viewed both the target and the cursor throughout each trial, but shortly before interception, the cursor motion became temporarily decoupled from optical mouse movements. Thus, perception of the target trajectory remained intact, whereas late corrective motor commands could no longer influence cursor movement.

To test this idea, participants performed a validated visuomotor interception task in which a horizontally controlled cursor intercepted a gravity-like falling target (Márquez et al., 2024). Manual and gaze trajectories were analyzed continuously relative to both the target’s instantaneous position and its predicted landing position, allowing us to quantify when behavior became committed to the future interception location. We hypothesized that preventing effective late online corrections would redistribute control toward earlier predictive planning. Specifically, if predictive and feedback-based control represent complementary components of a common control policy, eliminating the opportunity for late corrections should produce earlier convergence of manual behavior toward the predicted landing position.

## Methods

### Participants

Sixty undergraduate students (30 female) participated voluntarily in the study and provided written informed consent in accordance with the Declaration of Helsinki and institutional guidelines. The mean age was 21.39 ± 0.19 years (modal age = 21). Sample size was determined *a priori* based on effect sizes reported in previous visuomotor interception studies, with the power analysis incorporating an expected 70% yield of valid eye-tracking recordings (Márquez et al., 2024; Mrotek and Soechting, 2007). All participants completed a standardized 62-item questionnaire assessing sociodemographic variables, health status, daily habits, and academic background (Treviño et al., 2024). Participants reported no history of psychiatric, neurological, or neurodevelopmental disorders. Normal or corrected-to-normal visual acuity was confirmed using a standard Snellen chart assessment. The study protocol was approved by the Ethics Committee of the Instituto de Neurociencias, Universidad de Guadalajara (reference: ET102021-330).

### Experimental setup

Experimental sessions lasted approximately 55 min and were conducted in a dimly lit room. Participants were seated comfortably at a viewing distance of 70 cm from a 24-inch LCD monitor (refresh rate: 60 Hz; resolution: 1920 × 1,080 pixels), subtending 52.7° × 30.4° of visual angle. Head position was stabilized using a chin–forehead rest to minimize head movement. Manual responses were collected using a wired optical mouse positioned centrally below the screen. Auditory feedback was delivered binaurally through standard over-ear headphones. All stimulus presentation, data acquisition, and analysis were implemented in MATLAB 2025b with Psychophysics Toolbox (MathWorks, Natick, MA).

### Visuomotor interception task

Participants performed a validated visuomotor interception task designed to probe predictive control during continuous sensorimotor behavior (Márquez et al., 2024). Using the optical mouse, participants controlled a horizontal blue cursor (2° width × 0.3° height of visual angle) constrained to move along the horizontal axis at the bottom of the screen. The cursor served as an interception effector.

On each trial, a black circular target (0.3° diameter) appeared at the upper center of the screen and remained stationary during a 300-ms countdown period. The cursor began each trial from its final position in the preceding trial, preserving continuity of manual state across successive trials. Trials began automatically following completion of the preceding trial. After the countdown, the target followed a downward parabolic trajectory governed by a constant vertical acceleration, an initial horizontal velocity (leftward or rightward), and a quadratic drag term that emulated air resistance, a retarding force opposing motion and growing with the square of speed (Márquez et al., 2024). The vertical component was set by a single constant acceleration, identical across trials, so every fall lasted 4.17 s (250 frames at 60 Hz); only the initial horizontal velocity varied, and it alone generated the spread in landing positions. Initial horizontal velocity was sampled uniformly over −4.23 to +4.23 deg./s, and landing positions spanned 52.5° of visual angle. This was deliberate. Fixing the fall time removed the temporal degree of freedom: participants never had to estimate time-to-contact, and the task reduced to a problem of spatial commitment. Response timing was therefore not a dependent variable; the RMSE metrics (described below) index spatial anticipation, not time-to-contact.

**Figure 1:**
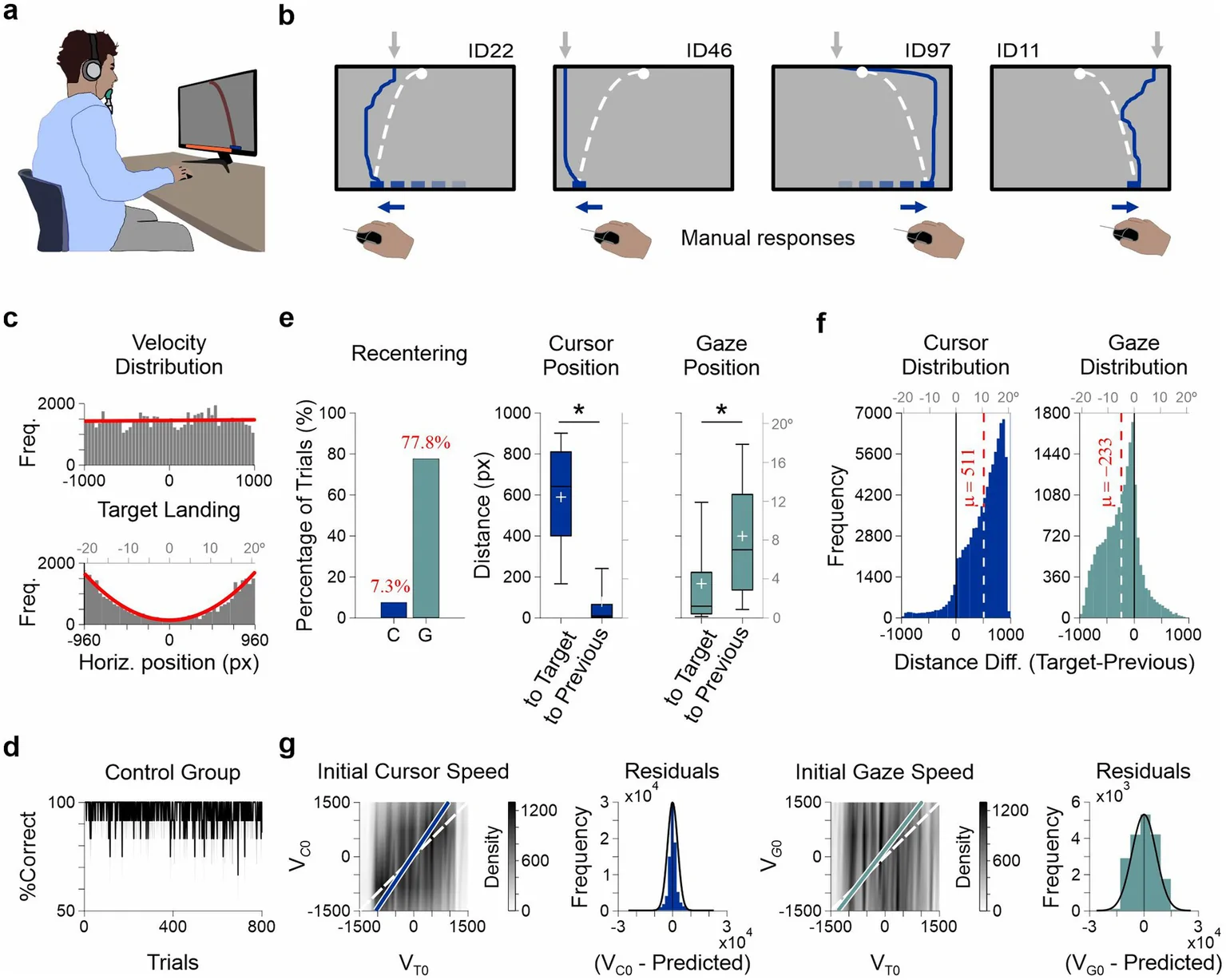
Task structure and baseline manual and oculomotor behavior. **(a)** Schematic of the visuomotor interception task. Participants controlled a horizontal cursor with an optical mouse to intercept a target moving downward along a gravity-like parabolic trajectory. **(b)** Example control trials showing target trajectories (white dashed lines) and the corresponding horizontal cursor trajectories (blue), projected onto the vertical axis. **(c)** Target kinematics. Upper: distribution of initial horizontal target velocities (V_T0_; range: −4.23 to +4.23 deg./s), showing uniform sampling across trials. Lower: resulting distribution of target landing positions, which is U-shaped because of the task dynamics. **(d)** Mean interception performance across the experimental session under control conditions. **(e)** Trial-onset recentering analysis. Left: proportion of trials in which the initial cursor (C) or gaze (G) position was closer to the initial target position than to the previous trial’s endpoint. Middle and right: distances from the initial cursor and gaze positions to the initial target position and to the previous endpoint. **(f)** Distributions of distance differences (distance to the initial target position minus distance to the previous endpoint) for the cursor (left) and the gaze (right). Positive values indicate greater proximity to the previous endpoint, whereas negative values indicate recentering toward the initial target position. Secondary axes in **c,e,f** show the same distances in degrees of visual angle. **(g)** Initial cursor (V_C0_) and gaze (V_G0_) velocities as a function of V_T0_ (negative values indicate leftward motion and positive values indicate rightward motion). The white dashed line indicates unity (slope = 1); slopes greater than one indicate response velocities that exceed the target velocity. Asterisks mark significant paired comparisons between distances (paired t-tests, Holm–Bonferroni corrected; *p* < 0.001).

Participants were instructed to move the cursor to intercept the target at its landing position at the bottom of the screen. Each block comprised 50 trials; the first 10 were used for familiarization and excluded from all analyses, leaving 40 analyzed trials per block. Each participant completed 10 blocks (500 trials total, 400 analyzed): five control blocks and one block at each of the five freeze durations. Performance feedback was provided at the end of each trial via simultaneous visual and auditory cues, each lasting 100 ms. Visual feedback consisted of a full-screen color change (dark earthy green for successful interceptions and dark red for unsuccessful ones). Auditory feedback was delivered via a 100-ms pure tone; successful trials were signaled by a 10 kHz tone and unsuccessful trials by a 1 kHz tone, following established procedures (Márquez et al., 2024). Task structure and example trials are shown in Figures 1a,b.

### Cursor-freezing manipulation

To transiently disrupt online visuomotor control while preserving visual input, we implemented a cursor-freezing manipulation in which the cursor remained visible but became temporarily unresponsive to mouse movements immediately before target landing. Only the mapping between mouse displacement and cursor motion was interrupted; the visual display of both cursor and target remained unchanged throughout the trial. Mouse movements were still recorded during the frozen interval, allowing manual behavior to be analyzed independently of cursor motion. Freezing was applied during the final 10, 20, 30, 40, or 50 frames of target motion, corresponding to 167, 333, 500, 667, or 833 ms before landing. Because target trajectories were identical across conditions, any between-condition differences reflect only the duration of unavailable late motor correction.

Freezing durations were presented in separate blocks, with the order counterbalanced across participants and either ascending or descending. We used a blocked design because our goal was not to measure immediate responses to an unexpected perturbation, but to characterize the control policy adopted under a sustained reduction in late motor controllability. Keeping freezing duration constant within each block also allowed us to assess gradual behavioral adjustment with trial-by-trial regression analyses. Participants were told only that some blocks would differ from baseline and were not informed of the freezing duration. Freezing and non-freezing control blocks were interleaved within each session, providing within-subject baseline measurements under the same visual conditions. Each session contained 10 blocks: five control blocks and five freezing blocks, one for each freezing duration. Control and freeze blocks alternated, so each freeze block was immediately preceded and followed by a control block from the same session segment. For each freeze duration, the control baseline used in the comparisons was the mean of the two adjacent control blocks. This adjacency rule matched session position, fatigue, and eye-tracker drift across compared conditions. Analyses using the pooled control blocks rather than the adjacent ones yielded the same qualitative pattern, with cluster onsets differing by no more than 2 frames.

### Eye-tracking calibration and acquisition

Binocular eye movements were recorded using a Tobii Pro Fusion eye tracker (Tobii Pro SDK for Windows; Tobii AB, Stockholm, Sweden). Participants’ head position was constrained throughout the experiment to maintain stable tracking quality. Eye-tracking accuracy was established using a two-step calibration procedure performed at the beginning of each experimental block. First, a standard four-point calibration was conducted using the manufacturer’s software. This was followed by a custom validation routine implemented in the Psychophysics Toolbox, which required fixation on four peripheral targets spanning the display area (Márquez and Treviño, 2024a). Eye movements were sampled at 60 Hz, providing one gaze estimate per display frame (16.7 ms). The four-point calibration procedure, together with the viewing geometry, yielded a nominal spatial resolution of approximately 0.26°, comparable to the target’s diameter (0.3°). Across participants, the average proportion of valid gaze samples was 81.5% (minimum 57.5%; Supplementary Figure 1). A gaze sample was considered valid whenever the eye tracker returned a horizontal gaze estimate for that display frame. For each participant, gaze validity was computed as the proportion of valid samples relative to the total number of recorded samples across all analyzed trials (excluding familiarization trials) within the 250-frame analysis window.

The experiment was divided into two halves separated by a brief rest period. Eye-tracker calibration was repeated at the beginning of the second half and whenever necessary during the experiment. Blink-related signal losses were corrected using linear interpolation (Márquez and Treviño, 2024b). Binocular gaze position was computed as the average of both eyes. Participants received no explicit gaze instructions to preserve spontaneous oculomotor behavior.

### Data analyses

Manual and oculomotor behavior were quantified using four reference-frame-specific root mean square error (RMSE) measures. The first letter identifies the effector (C, cursor; G, gaze), whereas the second identifies the reference frame (C, the target’s instantaneous position; L, the target’s final landing position). Consequently, RMSE_CC_ and RMSE_GC_ quantify tracking accuracy relative to the target’s current position, whereas RMSE_CL_ and RMSE_GL_ quantify alignment with its predicted landing location. All four metrics were computed frame-by-frame throughout the entire target fall. Analyses were performed separately for successful and unsuccessful trials and, for cursor-freezing comparisons, were restricted to the pre-freeze interval (see below).

These RMSE measures quantify tracking error across complementary spatial reference frames rather than directly measuring predictive or feedback-based control. A low RMSE relative to the target’s current position, for example, does not necessarily imply feedback-dominated behavior, because a highly predictive movement may transiently coincide with the moving target. Instead, the balance between predictive planning and online correction is reflected in the temporal evolution of the two RMSE trajectories. Earlier reductions in landing-referenced error indicate earlier commitment to the future interception location, whereas sustained reductions in target-referenced error reflect continuous tracking of the target’s ongoing motion. Thus, predictive and feedback-based control were inferred indirectly from the relative timing of these behavioral signatures rather than from their absolute magnitudes. Rather than assuming this interpretation, we evaluated it independently using a simulation with known ground-truth control policies (see Validation of the RMSE metrics).

Cursor and gaze positions were converted from pixels to degrees of visual angle using the exact display geometry and an arctangent transformation, which accounts for the dependence of pixel-to-degree conversion on retinal eccentricity. Averaged across the display this corresponds to 36.40 px/deg., rising to 33.80 px/deg. at screen center. All statistical analyses were performed using these exact sample-wise conversions. For visualization only, degree axes shown in the figures were generated using the corresponding linear conversion at screen center because geometric deviations over the illustrated range were negligible.

Manual movement onset was defined as the first frame in which cursor displacement exceeded 5 pixels (0.148°) relative to its starting position. As a validation, we compared this spatial criterion with a conventional velocity threshold of 30°/s and found agreement in 96% of trials, indicating that both definitions identified movement onset equivalently. Oculomotor onset was defined as the first frame at which gaze velocity exceeded 50°/s. To distinguish immediate behavioral reorganization from progressive within-block adaptation, each experimental block was divided into its first and last 20 analyzed trials. Interception accuracy was quantified separately for both windows, allowing early responses to a newly introduced freezing condition to be compared with behavior after participants had adopted a stable control policy. Unless otherwise stated, averaged RMSE trajectories were computed from the last 20 trials of each block because these best represent steady-state behavior under each experimental condition. This restriction was adopted to characterize behavior after participants had experienced the manipulation. However, the conclusions did not depend on this choice. Trial-by-trial regression analyses showed that earlier predictive commitment was already detectable in the first trials of each freezing condition and remained stable thereafter. A similar qualitative pattern was obtained when all 40 analyzed trials were included.

Although each RMSE metric quantifies alignment with a reference frame, direct comparisons between the current-position and landing-position errors provide a more intuitive measure of the balance between online tracking and predictive commitment. We therefore computed four complementary difference indices.

For manual behavior, the absolute difference index was defined as


$$\Delta (t)=\mathrm{RMS}E_{\mathrm{CL}}(t)-\mathrm{RMS}E_{\mathrm{CC}}(t)$$ (1)

Positive values indicate that the cursor was closer to the target’s instantaneous position than to its predicted landing position, whereas negative values indicate greater alignment with the future interception point than with the moving target itself. Thus, progressively more negative values reflect earlier commitment toward the predicted landing location.

Because *Δ*(t) depends on the absolute magnitude of the RMSE values, we also computed a normalized index,


$$I(t)=\frac{\mathrm{RMS}E_{\mathrm{CL}}(t)-\mathrm{RMS}E_{\mathrm{CC}}(t)}{\mathrm{RMS}E_{\mathrm{CL}}(t)+\mathrm{RMS}E_{\mathrm{CC}}(t)}$$ (2)

which expresses the same relationship independently of the overall error scale. This normalization constrains the index between −1 and +1 and reduces the influence of between-condition differences in absolute tracking error.

Equivalent indices were computed for gaze:


$$G(t)=\mathrm{RMS}E_{\mathrm{GL}}(t)-\mathrm{RMS}E_{\mathrm{GC}}(t)$$ (3)

and


$$I_{G}(t)=\frac{\mathrm{RMS}E_{\mathrm{GL}}(t)-\mathrm{RMS}E_{\mathrm{GC}}(t)}{\mathrm{RMS}E_{\mathrm{GL}}(t)+\mathrm{RMS}E_{\mathrm{GC}}(t)}$$ (4)

These indices provide direct within-effector comparisons between current-position and landing-position alignment, allowing changes in predictive commitment to be evaluated independently of overall tracking accuracy.

### Statistical methods

Interception accuracy across control and freezing conditions was analyzed using one-way repeated-measures ANOVA, with freeze duration as the within-subject factor. Significant effects were followed by paired *t*-tests comparing control and freezing conditions. When the assumptions of the paired *t*-test were not met, the Wilcoxon signed-rank test was used instead. ANOVA results are reported as F, *p*, and partial *η^2^*, and paired comparisons as *t*, degrees of freedom, *p*, and Cohen’s *d*. Time-resolved RMSE trajectories were compared using cluster-based permutation tests, which control the family-wise error rate across multiple time points (Maris and Oostenveld, 2007). At each frame, paired *t*-tests were computed across participants. Clusters were defined as runs of at least three consecutive frames with |t| exceeding the two-tailed critical value (*p* < 0.05). Cluster statistics were computed as the sum of t values within each cluster and evaluated against a null distribution generated from 5,000 random sign-flips of the paired differences. For each significant cluster, we report the cluster-level *p* value, onset and offset times, and the peak Cohen’s *d*. Cluster-based tests establish that a difference exists somewhere within a cluster, but they do not identify the exact onset of that effect, and cluster boundaries should not be compared directly across conditions (Sassenhagen and Draschkow, 2019). For that reason, cluster limits are reported descriptively only. When the timing of an effect was itself the main claim, we estimated onset at the participant level with a change-point fit to the individual difference trace and tested those onsets directly.

To assess the overall effects of the experimental factors before conducting specific *post hoc* comparisons, average RMSE values within the pre-freeze interval were analyzed using linear mixed-effects models. The primary model included Condition (control and five freezing durations), Epoch (first versus last 20 trials), and Effector (cursor versus gaze) as fixed effects, together with all interactions, and subject-specific random intercepts. A companion model, restricted to manual behavior, replaced Effector with Reference Frame (current versus landing position) to directly compare the two spatial reference frames. Model residuals were evaluated using the Shapiro–Wilk test. When residuals deviated from normality, RMSE values were Box–Cox transformed before refitting the model; this transformation did not alter the statistical conclusions. Multiple paired comparisons performed outside the cluster-based permutation framework, including control-versus-freeze accuracy comparisons and trial-onset recentering analyses, were corrected using the Holm–Bonferroni procedure. Unless otherwise stated, statistical analyses were performed on participant-level averages. Data are reported as mean ± SEM, and all tests were two-tailed with *α* = 0.05.

## Results

### Baseline visuomotor behavior reveals stable manual trajectories and trial-by-trial gaze recentering

Participants performed a visuomotor interception task in which horizontal mouse movements controlled a cursor to intercept a falling target at its predicted landing position (Figure 1a). Target motion followed a parabolic trajectory determined by an initial horizontal velocity, constant downward acceleration, and a velocity-dependent damping term (Márquez et al., 2024). Sample trajectories showed continuous cursor adjustments throughout the fall, indicating sustained engagement with target motion (Figure 1b).

Initial horizontal velocities were sampled uniformly. Because the fall duration was fixed, the initial horizontal velocity uniquely determined the target’s landing position. Negative values corresponded to leftward motion and positive values to rightward motion. Throughout this manuscript, target velocity refers to its horizontal component (the initial vertical velocity was zero). The empirical distribution of horizontal velocities was uniform (upper panel, Figure 1c) and was well described by a linear fit with a negligible slope (𝑦 = 0.22𝑥 + 1,500; 𝑅^2^ = 0.004; RMSE = 189.2). Integrating velocity over the fixed fall duration yielded landing positions. Although initial horizontal velocities were sampled uniformly, the quadratic drag term compressed higher velocities more strongly because the retarding force increased with the square of speed. Consequently, a wider range of initial velocities converged onto increasingly similar landing positions, increasing the density of trajectories near the lateral edges of the screen and producing a symmetric U-shaped spatial distribution (lower panel, Figure 1c). Accordingly, this distribution was well described by a quadratic function (𝑦 = 0.0033𝑥^2^–6.4𝑥 + 3,700; 𝑅^2^ = 0.981; RMSE = 126.7). Thus, the spatial distribution emerged from the task dynamics rather than from the velocity sampling procedure.

Baseline interception performance remained stable throughout the session, with no sign of fatigue or learning (Figure 1d). We next examined whether the cursor and gaze reset between consecutive trials by comparing their initial positions with both the current target position and the previous trial’s endpoint. Cursor position showed little recentering: only 7.3% of trials began closer to the current target than to the previous cursor endpoint (Figure 1e). In contrast, gaze showed strong recentering, with 77.8% of trials beginning closer to the new target than to the previous gaze endpoint.

The initial cursor–target distance was 580 ± 48 pixels, substantially larger than the distance to the previous cursor endpoint (77 ± 34 pixels; *t*_(59)_ = 11.4, *p* < 0.001, *d* = 1.47). Conversely, gaze started closer to the current target (167 ± 42 pixels) than to the previous gaze endpoint (399 ± 59 pixels; paired *t*_(59)_ = −5.35, *p* < 0.001, *d* = −0.69). The proportion of recentering trials differed between cursor and gaze (χ^2^_(1)_ = 56, *p* < 0.001). Distance-difference distributions (the difference between the current target distance and the previous endpoint distance) confirmed this pattern (Figure 1f). Cursor values were predominantly positive, indicating persistence from the previous trial, whereas gaze values were predominantly negative, indicating recentering toward the new target. A secondary axis above these panels shows the same distances in degrees of visual angle.

We next asked whether the initial cursor and gaze responses reflected target motion. Movement onset was defined as the first frame in which the cursor position changed by at least five pixels (0.148°), and initial velocity was computed over a 5-frame (~80 ms) window. The same procedure was applied to gaze. Initial cursor and gaze velocities both increased with the target’s initial horizontal velocity (Manual: 𝑦 = 1.492 𝑥 + 63.12; 𝑅^2^ = 0.112; RMSE = 2811.46; 𝑟 = 0.335; Gaze: 𝑦 = 1.294 𝑥 + 194.4; 𝑅^2^ = 0.018; RMSE = 6669.81; 𝑟 = 0.136; first and second panels, Figure 1g). Negative and positive values corresponded to leftward and rightward target motion, respectively. In both regressions, the slope exceeded one, indicating that the initial responses were faster than target motion, consistent with temporal and biomechanical compensation (Márquez and Treviño, 2026).

Although the regression slopes were similar for cursor and gaze, their explanatory power differed markedly. Target velocity explained 11.2% of the variance in initial cursor velocity but only 1.8% of the variance in initial gaze velocity (*R*^2^ = 0.112 versus 0.018), indicating that manual responses exhibited a much more consistent trial-by-trial relationship with target motion. Consistent with this result, both cursor and gaze reduced spatial error after movement onset (second and fourth panels, Figure 1g), although gaze remained substantially more variable, as expected for oculomotor tracking. Manual behavior therefore yielded a much stronger population-level signature after averaging, whereas gaze variability reduced the detectability of comparable effects.

### Validation of the RMSE metrics

**Figure 2:**
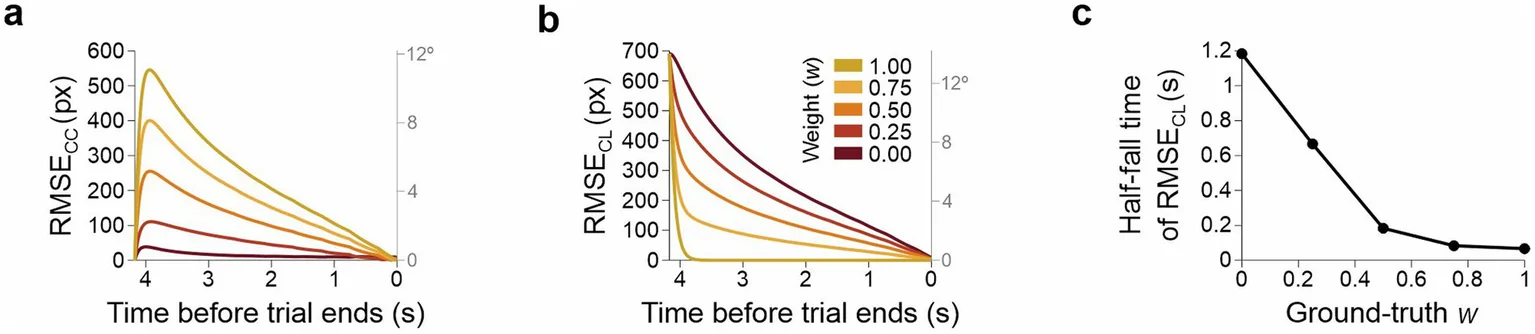
A mixture-controller simulation validates RMSE_CC_ and RMSE_CL_ as diagnostic metrics of feedback versus predictive control. **(a)** Simulated RMSE_CC_ traces across five mixture weights (*w* = 0, 0.25, 0.5, 0.75, 1). A first-order controller was driven toward ref(t) = (1 − *w*) · target(*t*) + *w* · landing, with *τ* = 83 ms, and fed empirical target trajectories (500 synthetic trials per weight). τ was fixed rather than fitted, and set deliberately below the latency of visually guided manual correction, which begins roughly 100–150 ms after a target displacement (Oostwoud Wijdenes et al., 2011), so that the simulated controller is never slower than the system it stands in for; at *w* = 1, this choice reproduces the analytic half-fall time of 0.07 s. Pure feedback (*w* = 0) tracks the moving target throughout the trial; pure predictive control (*w* = 1) sits at the landing position and converges only near the end. **(b)** Simulated RMSE_CL_ traces for the same five weights. All conditions start at the same initial value but diverge in how quickly the trace decays. Higher *w* produces an earlier drop. Color indexes *w*. **(c)** Half-fall time of RMSE_CL_ as a function of the ground-truth weight *w*. The relationship is strictly monotonic (Spearman *r* = −1.00), confirming that the timing of the RMSE_CL_ decline recovers the underlying control regime.

To quantify the temporal evolution of manual control, we used two complementary root-mean-square error (RMSE) measures that describe alignment with either the target’s instantaneous position (RMSE_CC_) or its predicted landing position (RMSE_CL_). Before analyzing the experimental data, we validated whether these temporal profiles could distinguish feedback-dominated from predictive control. We simulated a simple one-parameter control model that interpolated between tracking the target’s current position (*w* = 0) and moving directly toward its landing position (*w* = 1), using each participant’s actual target trajectories to generate synthetic cursor traces. Under pure feedback control (*w* = 0), RMSE_CC_ remained low throughout the trial (Figure 2a), whereas RMSE_CL_ decreased only near target landing (Figure 2b), when the current and landing positions converged. Under fully predictive control (*w* = 1), RMSE_CL_ decreased immediately and remained low, whereas RMSE_CC_ stayed elevated because the cursor moved directly toward the predicted landing position instead of following the moving target. Intermediate controller weights produced intermediate temporal profiles.

To quantify these differences, we measured the half-fall time of RMSE_CL_, defined as the time required for the trace to reach half of its initial value. This measure varied monotonically across controller weights (Spearman *r* = −1.00 across *w* = 0, 0.25, 0.5, 0.75, 1; half-fall times: 1.18, 0.67, 0.18, 0.08, and 0.07 s, respectively; Figure 2c). Thus, the temporal profile of RMSE_CL_ recovered the simulated control regime. Sensitivity, however, was not uniform across the full range of controller weights. Simulations using a finer grid (*w* = 0:0.05:1; 500 synthetic trials per weight) showed that the local sensitivity (|dt½/d*w*|) declined below 20% of its maximum for weights above ~0.40, indicating reduced resolution in the highly predictive range. To determine whether this limitation affected our data, we mapped each empirical RMSE_CL_ half-fall time onto the simulated curve. All experimental conditions corresponded to estimated weights below *w* = 0.2, well within the steep, high-sensitivity region of the mapping (Supplementary Figure 2).

The estimated weights should not be interpreted as the participants’ actual balance between predictive and feedback control because the simulation was calibrated using an idealized noiseless controller. Instead, the inversion establishes the relative ordering of conditions along a monotonic predictive-control axis. A second independent measure, the area between the RMSE_CC_ and RMSE_CL_ curves, produced a similar monotonic ordering across the entire range of controller weights (Spearman *r* = 1.00) and showed a more uniform sensitivity profile (flatness CV = 0.22 versus 1.16; Supplementary Figure 2). Together, these analyses indicate that the temporal evolution of the RMSE curves captures changes in the balance between predictive and feedback control.

### Manual and gaze behavior across trial outcomes

**Figure 3:**
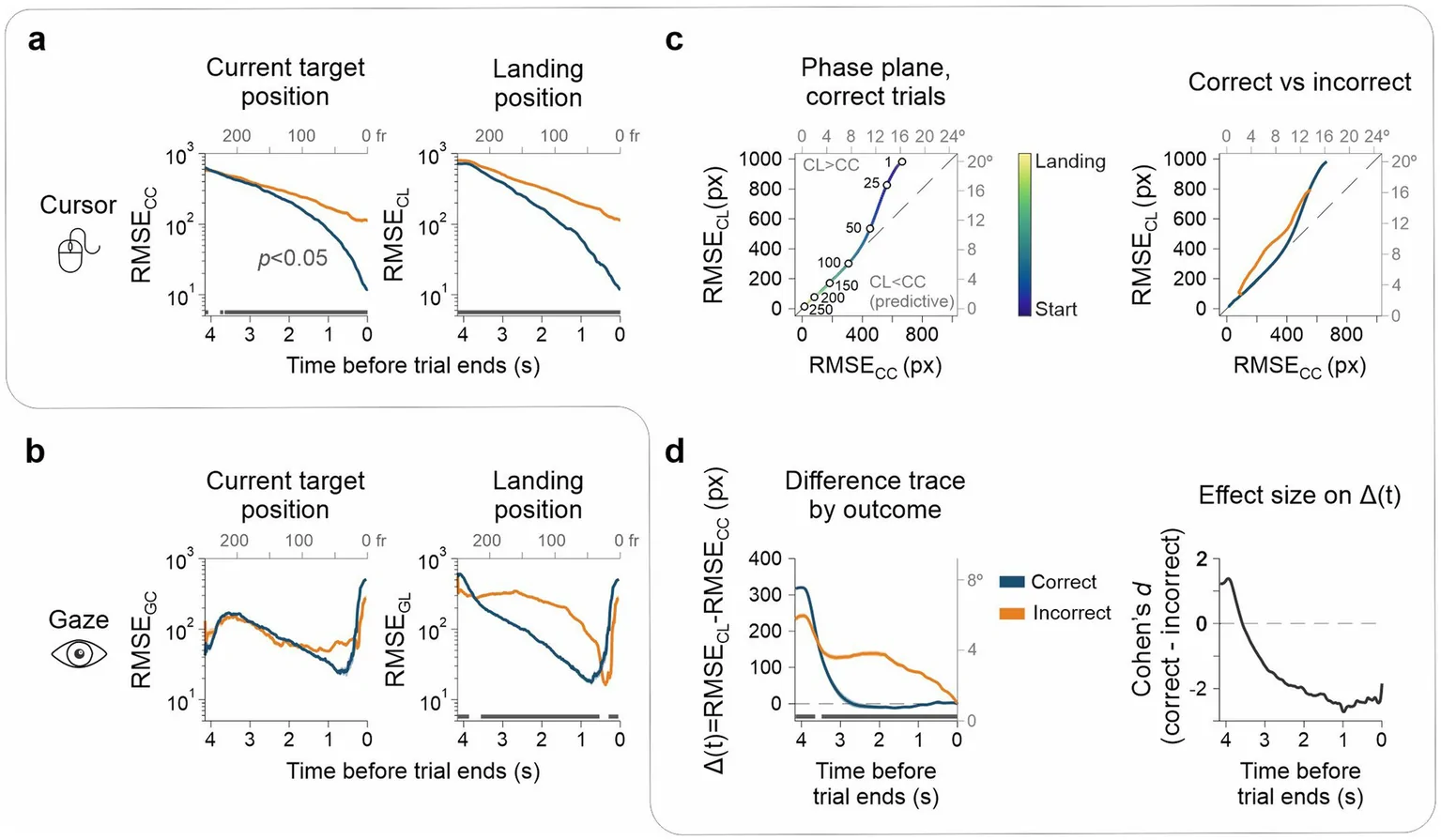
Reference-frame-specific RMSE dissociates predictive and feedback-based components of manual and gaze behavior. **(a,b)** Time-resolved RMSE trajectories for correct (dark blue) and incorrect (orange) trials. Panels are organized by effector (rows) and reference frame (columns). The top row shows manual (cursor; C) behavior and the bottom row gaze (G). The left column shows RMSE relative to the target’s current position (RMSE_CC_, RMSE_GC_), whereas the right column shows RMSE relative to the target’s final landing position (L; RMSE_CL_, RMSE_GL_). The upper x-axis indicates time expressed as frames before trial termination. Horizontal bars below each panel indicate significant clusters identified by cluster-based permutation tests (cluster-level *p* < 0.05, 5,000 permutations). **(c)** Left: phase-plane representation of the manual trajectories (RMSE_CL_ versus RMSE_CC_). The dashed identity line (RMSE_CL_ = RMSE_CC_) marks equal error relative to the current and landing positions; its intersection with the trajectories indicates when landing-referenced error becomes smaller than current-referenced error. Right: phase-plane trajectories for correct (dark blue) and incorrect (orange) trials. **(d)** Left: difference trace, *Δ*(t) = RMSE_CL_ − RMSE_CC_, for correct (dark blue) and incorrect (orange) trials. Negative values indicate that the cursor was closer to the target’s landing position than to its current position. Right: time-resolved effect size (Cohen’s *d*) for the difference between correct and incorrect Δ(t) traces.

To characterize manual and oculomotor behavior during target interception, we quantified the RMSE between each effector (cursor, ‘C’, or gaze, ‘G’) and the target throughout the trial in two reference frames: the target’s current position (RMSE_CC_ and RMSE_GC_) and its predicted landing position (RMSE_CL_ and RMSE_GL_; Figure 3). Temporal differences between conditions were evaluated using cluster-based permutation tests, which identified contiguous time periods during which the RMSE trajectories differed. Significant clusters are shown below each RMSE trace in Figure 3.

Manual behavior differed markedly between successful and unsuccessful interceptions in both reference frames. For RMSE_CC_, successful trials showed an early period of higher error (0.02–1.23 s; sum-*t* = +540.0, cluster *p* = 0.0004, peak |*t*_(59)_| = 17.12, peak *d* = +2.21), followed by a prolonged period of lower error from 1.98 s until target interception (sum-*t* = −726.5, *p* = 0.0002, peak |*t*_(59)_| = 10.36, peak *d* = −1.34). For RMSE_CL_, successful trials also showed an early increase in error (0.02–0.68 s; sum-*t* = +464.6, *p* = 0.0100, peak |*t*_(59)_| = 18.51, peak *d* = +2.39), followed by a sustained reduction in landing-position error from 0.93 s until the end of the trial (sum-*t* = −2747.0, *p* = 0.0002, peak |*t*_(59)_| = 21.47, peak *d* = −2.77), indicating earlier convergence toward the predicted interception point (Figure 3a).

Gaze showed a different pattern. No significant clusters were detected for RMSE_GC_ (largest cluster *p* = 0.23), indicating that our analyses did not detect a reliable population-level difference in instantaneous target tracking between successful and unsuccessful trials. In contrast, RMSE_GL_ showed a single late cluster between 3.28 and 3.77 s (sum-*t* = −598.0, cluster *p* = 0.0020, peak |*t*_(59)_| = 20.99, peak *d* = −2.71), with lower landing-position error during successful trials (Figure 3b). In the same participants, the corresponding manual effects extended until target interception (RMSE_CC_: 3.63–4.17 s, sum-*t* = −213.8, *p* = 0.0088, peak |*t*_(59)_| = 21.25, *d* = −2.74; RMSE_CL_: 3.58–4.17 s, sum-*t* = −325.4, *p* = 0.0002, peak |*t*_(59)_| = 21.72, *d* = −2.80). Thus, manual and gaze responses differentiated successful from unsuccessful trials at different times. Gaze reflected the trial outcome by aligning with the predicted landing position during the final ~0.5 s of the trial, whereas manual trajectories separated the two outcomes over a much longer stretch of the movement, up to interception.

To determine whether the reduction in RMSE_CL_ reflected predictive behavior rather than a general reduction in tracking error, we compared RMSE_CC_ when RMSE_CL_ reached half of its initial value (not illustrated). On successful trials, RMSE_CC_ still retained 62.3 ± 0.6% of its initial value, significantly above the 50% value reached by RMSE_CL_ (*t*_(59)_ = 21.24, *p* < 0.001; median RMSE_CL_ half-fall time = 0.94 s). Thus, landing-position error decreased faster than instantaneous tracking error, indicating earlier commitment toward the future interception point. A similar conclusion emerged from the phase-plane analysis. The group-average trajectory crossed the identity line (RMSE_CL_ = RMSE_CC_) at 1.48 s, when RMSE_CC_ still retained 50.7% of its initial value (Figure 3c). Likewise, the difference between the two metrics, *Δ*(t) = RMSE_CL_ − RMSE_CC_ (Equation 1), distinguished successful from unsuccessful trials in two significant clusters: an early positive cluster (0.02–0.52 s; sum-*t* = +254.6, *p* = 0.0364, peak *d* = +1.38, peak |*t*_(59)_| = 10.69) followed by a prolonged negative cluster (0.68–4.17 s; sum-*t* = −3204.2, *p* = 0.0002, peak *d* = −2.72, peak |*t*_(59)_| = 21.07), reflecting progressively stronger alignment with the predicted landing position during successful interceptions (Figure 3d).

### Cursor freezing produces a graded loss of interception accuracy

We next examined how transient decoupling of hand movements from cursor motion reorganized interception behavior. During freezing trials, the cursor remained visible but became unresponsive to mouse movements for a fixed interval immediately before target interception. Five freezing durations were tested: 10, 20, 30, 40, and 50 frames, corresponding to 167, 333, 500, 667, and 833 ms, respectively.

**Figure 4:**
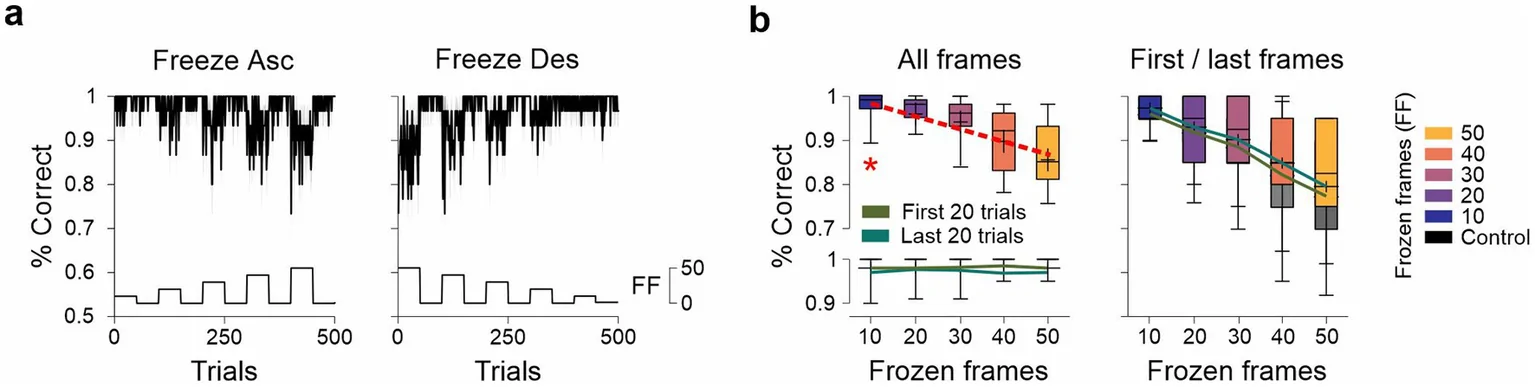
Cursor freezing results in a graded loss of interception accuracy. **(a)** Mean percentage of successful interceptions across the experimental session for blocks with increasing (left) or decreasing (right) freezing durations. Freezing blocks are indicated by the vertical bars along the bottom of each panel; bar height maps freeze duration, not block index, so the two panels are directly comparable; the continuous trace shows group-averaged performance. **(b)** Mean interception performance as a function of freezing duration. The main panel plots accuracy against the five freeze durations (10–50 frames, i.e., 167–833 ms) together with the unfrozen control level (87.1 ± 0.7%); accuracy falls monotonically from 96.4% at 10 frames to 78.2% at 50 frames. The right panel splits each freezing block into its early (first 20 analyzed trials, light green) and late (last 20 trials, dark green) epochs; both epochs decline linearly with freeze duration at indistinguishable slopes (−0.004 in each case, *p* = 0.78 for the difference). The lower-left inset applies the same early/late partition to the adjacent control blocks. Symbols are group means ± SEM.

Interception accuracy was high under control conditions (87.1% ± 0.7%), but decreased as freeze duration increased (one-way repeated-measures ANOVA, *F*_(5, 295)_ = 71.23, *p* < 0.001, *η^2^_p_* = 0.55; Figure 4). Across the five freezing conditions, performance declined monotonically from 96.4% at 10 frames to 78.2% at 50 frames (*t*_(59)_ = 6.43, paired Cohen’s *d* = 0.83). This decline was well described by a linear relationship. Neither a piecewise regression with a free breakpoint (Δ*R*^2^ < 0.005), nor the addition of a quadratic term (*p* = 0.34), improved the fit.

To assess within-block adaptation to cursor freezing, each freezing block was divided into early (first 20 trials) and late (last 20 trials) epochs (light and dark green traces, right panel of Figure 4b). The corresponding control blocks were partitioned identically (lower-left inset of Figure 4b). Interception accuracy decreased linearly with freeze duration in both the early (slope = −0.004, *t* = −16.70, *p* < 0.001, *R*^2^ = 0.989) and late epochs (slope = −0.004, *t* = −15.91, *p* < 0.001, *R*^2^ = 0.988). The slopes did not differ significantly (*p* = 0.78), suggesting that the behavioral cost of cursor freezing was already present at the beginning of each block and remained relatively stable throughout the block.

### Cursor freezing shifts manual control toward earlier predictive commitment

We next examined how cursor freezing altered the evolution of manual and gaze errors from target release to interception. Unless otherwise stated, trajectory analyses were performed on the last 20 trials of each condition, representing behavior after participants had experienced the corresponding freezing manipulation.

To distinguish changes in overall error magnitude from changes in the temporal evolution of behavior, we combined linear mixed-effects models with frame-by-frame cluster-based permutation tests. The mixed-effects models tested mean RMSE values in the pre-freeze interval, whereas the cluster analyses identified when along the trajectory the effects emerged. In the omnibus model on pre-freeze RMSE (Condition × Epoch × Effector), Condition (*F*_(5, 840.8)_ = 3.58, *p* = 0.003), and Effector (*F*_(1, 840.1)_ = 66.3, *p* < 0.001) were significant, as was the Condition × Epoch interaction (*F*_(5, 840.1)_ = 3.08, *p* = 0.009). Thus, freezing altered pre-freeze error. The two effectors differed in overall accuracy, and the effect of freezing depended on whether behavior was measured in the first or the last 20 trials of a block. The Condition × Effector interaction was not significant (*F*_(5, 840.1)_ = 0.42, *p* = 0.84). Within the manual system, a companion model (Condition × Epoch × Reference Frame) showed a significant three-way interaction (*F*_(5, 632.1)_ = 2.39, *p* = 0.036) and a strong Epoch × Reference Frame interaction (*F*_(1, 632.1)_ = 13.3, *p* < 0.001), indicating that freezing affected the time course of RMSE differently in the two reference frames. A four-way omnibus model that additionally included Reference Frame, fitted on the participants with simultaneous cursor and gaze recordings, reproduced the same pattern: Effector, Reference Frame, Epoch, and the Effector × Reference Frame interaction were all significant, whereas the higher-order terms were not. That four-way model, together with its residual diagnostics and comparisons against reduced and random-slope structures, is reported in Supplementary Figure 3.

**Figure 5:**
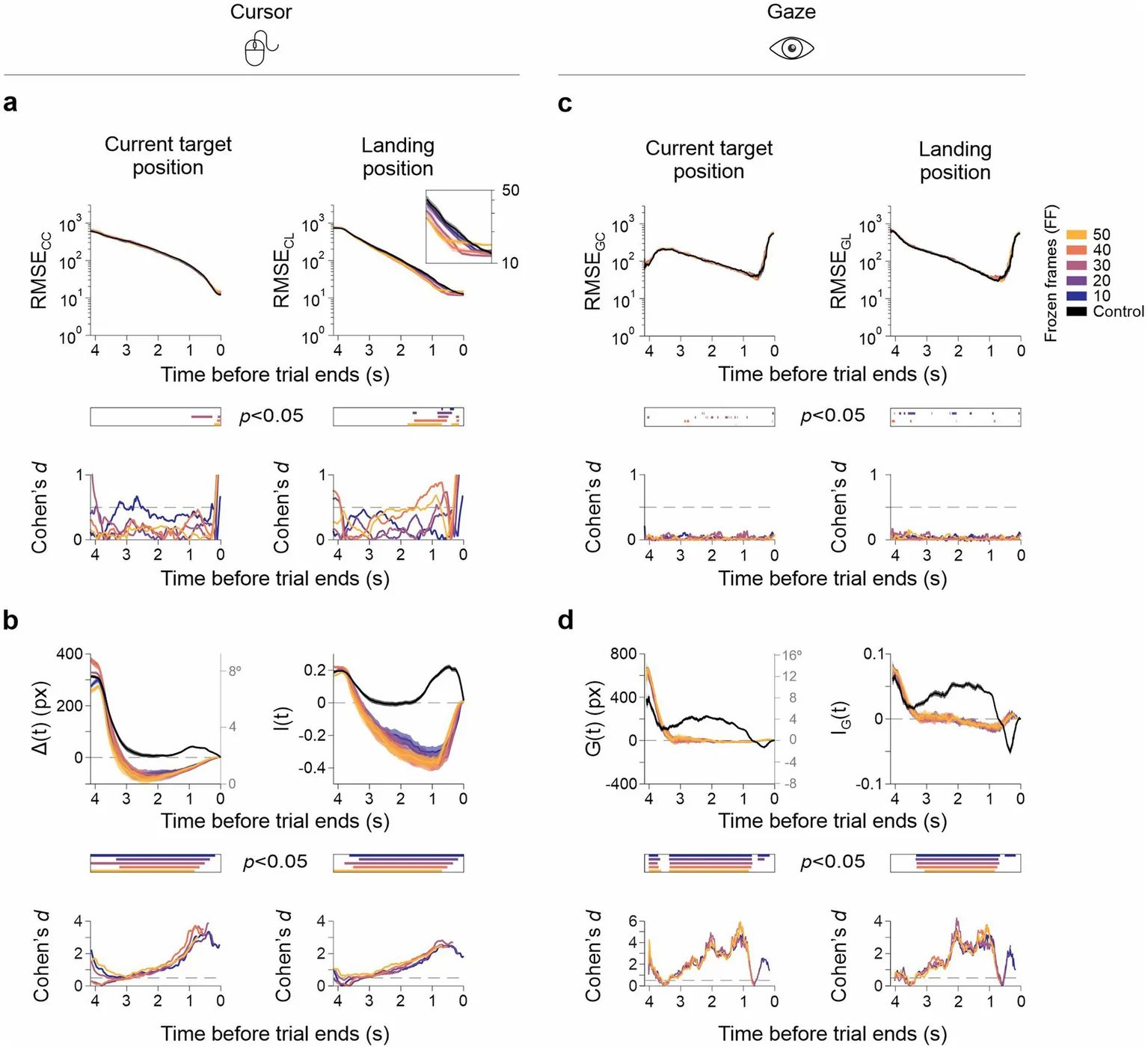
Transient disruption of visuomotor coupling reorganizes manual control toward the target’s future landing position. **(a)** Time-resolved RMSE traces for manual behavior, computed from the last 20 trials of each block. Left, RMSE relative to the target’s instantaneous position (RMSE_CC_), reflecting online tracking. Right, RMSE relative to the target’s landing position (RMSE_CL_), reflecting predictive alignment; traces are shown only up to freeze onset. Colored traces indicate freezing durations and the black trace the control condition. Horizontal bars denote significant clusters identified by cluster-based permutation tests (cluster *p* < 0.05). Lower panels show the absolute value of Cohen’s *d* (|*d*|) to facilitate comparison of effect magnitude across time. **(b)** Direct comparison of the two manual measures. Left, Δ(T) = RMSE_CL_ − RMSE_CC_. Right, normalized index, I(t) = (RMSE_CL_ − RMSE_CC_)/(RMSE_CL_ + RMSE_CC_). Negative values indicate greater alignment with the predicted landing position than with the target’s instantaneous position. **(c)** Time-resolved gaze RMSE traces, showing scattered clusters rather than the systematic shift seen for the hand. **(d)** Direct comparison of the gaze measures. Left, G(t) = RMSE_GL_ − RMSE_GC_. Right, normalized index, I_G_(t) = (RMSE_GL_ − RMSE_GC_)/(RMSE_GL_ + RMSE_GC_). Horizontal bars indicate significant clusters.

The cursor traces showed a consistent freezing-dependent reorganization of behavior (Figure 5a). RMSE_CC_ decreased relative to control in the terminal segment, with every freeze duration producing a significant negative cluster (peak *d* = −0.69 to −3.24). RMSE_CL_ followed a different trajectory. Relative to control, landing-referenced error first increased early in the trial and then fell below control before freeze onset in every freezing condition, yielding a biphasic pattern with an early positive cluster followed by a prolonged negative one (all cluster *p* ≤ 0.046; peak *d* = −2.2 to −3.1). Thus, when late visuomotor corrections were unavailable, the cursor became committed to the predicted landing position earlier in the trial.

To compare the two spatial reference frames directly, we examined the difference index *Δ*(t) = RMSE_CL_ − RMSE_CC_ (Equation 1) and its normalized counterpart I(t) (Equation 2) (Figure 5b). Relative to control, every freezing duration produced a prolonged negative cluster in both indices (all cluster *p* = 0.0002), indicating that cursor trajectories became aligned with the predicted landing position earlier in the movement. Peak effect sizes ranged from Cohen’s *d* = −3.07 to −3.87, with peak |*t*_(59)_| values between 23.78 and 29.98. The normalized index reproduced a similar temporal pattern, indicating that the observed shift toward earlier predictive commitment was independent of the overall magnitude of the RMSE measures. Because freezing durations were presented in blocks that either ascended or descended across the session, we asked whether the direction of the sequence contributed to this reorganization. Block order had no main effect on interception accuracy or on pre-freeze RMSE_CL_. The two sequences did, however, differ in how strongly pre-freeze RMSE_CL_ tracked freeze duration, with a reliable negative slope under the descending sequence and a zero slope under the ascending one (Supplementary Figure 4). The direction of the block sequence, therefore, modulated the sensitivity of the effect without producing it; the biphasic RMSE_CL_ pattern was present under both sequences.

An alternative account of this pattern could be that participants stopped predicting altogether and simply parked the cursor at a fixed, safe location once they knew a freeze was approaching. However, regressing the cursor position at freeze onset on the landing position of the same trial argues against this. The slope remained near unity in every freezing condition, exceeded the slope of the target’s own position on landing, and rose above the matched control frame rather than falling toward zero, so the cursor carried more trial-specific landing information than the display itself. Trial-specific prediction was favored over a fixed-parking model in every participant at every freeze duration, and the moment at which the cursor’s slope on landing overtook the target’s own occurred earlier under freezing than under control (Supplementary Figures 5a–e).

Gaze also changed under cursor freezing, but the changes were scattered rather than systematic (Figure 5c). RMSE_GC_ exhibited seven significant clusters across the five freezing durations, whereas RMSE_GL_ showed eight clusters whose timing and magnitude varied considerably across conditions. Unlike the stable reorganization observed in manual behavior, gaze responses did not exhibit a consistent shift toward either spatial reference frame. Consistent with this observation, the gaze difference index G(t) = RMSE_GL_ − RMSE_GC_ (Equation 3) and its normalized analog I_G_(t) (Equation 4) likewise failed to reveal any systematic change in the balance between target tracking and landing-position alignment across freezing conditions (Figure 5d). The omnibus model gave no evidence that freeze-dependent modulation differed between effectors (Condition × Effector, *F*_(5, 840.1)_ = 0.42, *p* = 0.84). Trial-averaged gaze pools tracking, prospective sampling, and monitoring of the hand into one trajectory, and the baseline analyses above already show that gaze carries a weaker population-level signature under averaging.

The normalized dissociation index separated hand and gaze during the second before freeze onset in every freeze condition tested: 3.00–4.00 s for the 10-frame freeze (cluster-based permutation test, *p* = 0.0006, peak *d* = +1.58), 2.93–3.83 s for 20 frames (*p* = 0.0004, *d* = +1.64), 2.93–3.67 s for 30 frames (*p* = 0.0064, *d* = +1.57), 2.85–3.50 s for 40 frames (*p* = 0.0112, *d* = +1.06), and 2.92–3.33 s for 50 frames (*p* = 0.0202, *d* = +0.75). The divergence began at a similar time across durations within the trial, suggesting that it was linked to trial time rather than to freeze duration itself. Gaze reached significance at roughly the same time in every condition, whereas the manual effect emerged earlier for the shorter freezes and later for the longer ones. We illustrate the reorganization of manual and gaze behavior in Supplementary Figure 6. Across representative participants, cursor trajectories progressively converged toward the predicted landing position before freezing onset, especially at the longest freezing durations.

Finally, we asked whether participants continued to move the mouse after cursor freezing began. Mouse displacement was recorded independently of cursor position, so it provides a measure of manual movement even when the cursor was locked. Integrated mouse displacement remained above zero at every freezing duration and increased approximately linearly with freeze duration (not illustrated). Within a block, however, integrated displacement during the freeze declined with trial number at every freeze duration (Supplementary Figure 5f), indicating that participants progressively reduced corrective movements after learning that the cursor no longer responded.

## Discussion

We investigated how predictive and feedback-based control were coordinated during visuomotor interception and how this balance changed when late motor corrections were selectively eliminated while visual information remained intact. Our cursor-freezing manipulation reduced interception accuracy and shifted manual behavior toward earlier commitment to the predicted landing position. Under normal conditions, successful interceptions were characterized by two complementary behavioral signatures: sustained alignment with the target’s instantaneous position together with progressively earlier convergence toward its future landing location. Rather than measuring feedback and prediction independently, complementary RMSE measures captured the temporal evolution of behavior in two reference frames, allowing changes in the relative contribution of predictive planning and online correction to be inferred throughout the movement. Together, these findings indicate that successful interception relied on a continuously updated control policy in which predictive planning and sensory feedback remained tightly integrated until target interception, consistent with previous accounts of visuomotor control (Brenner and Smeets, 2011, 2018; Márquez et al., 2024).

Gaze behavior showed a markedly different pattern from manual behavior. In our data, successful and unsuccessful trials differed primarily in gaze error relative to the target’s future landing position, whereas gaze error relative to the target’s instantaneous position showed little or no separation between outcomes. This dissociation suggests that gaze reflected the prediction of future interception locations more strongly than continuous target tracking, consistent with proposals that gaze supports the acquisition of information relevant for future actions rather than simply mirroring ongoing hand movements (Fooken et al., 2021; Land, 2006; Mrotek and Soechting, 2007; Treviño and Márquez, 2025; Zhao et al., 2019). Notably, this effect emerged only in the time-resolved analyses; conventional endpoint measures would have largely obscured it. Similar dissociations between oculomotor and manual behavior have been reported during covert and imagined interception tasks (D’Aquino et al., 2023).

Cursor freezing systematically reduced interception accuracy, and this reduction increased monotonically as freezing duration became longer. These findings directly quantify the contribution of late corrective commands to successful interception. Unlike visual occlusion paradigms, which simultaneously alter sensory information and motor behavior (Galea et al., 2011; Knelange and López-Moliner, 2020; Oudejans et al., 1996; Weber and Bellebaum, 2024), our manipulation preserved continuous visual access to both the target and the cursor while selectively preventing effective late motor corrections. We therefore interpret the observed performance deficit as arising primarily from the loss of corrective motor commands rather than from increased perceptual uncertainty or degraded sensory evidence. This distinction represents the principal conceptual difference between cursor freezing and previous occlusion-based paradigms (Oudejans et al., 1996), in which changes in sensory input and motor control are intrinsically coupled (Brenner and Smeets, 2011; Rothenberg-Cunningham and Newell, 2013). Our findings, therefore, show that, even in a task requiring substantial predictive control, successful interception continued to depend critically on the availability of late online corrections.

One aspect of the accuracy results warrants separate consideration. Although interception accuracy generally declined as freezing duration increased, the shortest freezing interval produced a small improvement relative to the control condition. This finding suggests that very brief disruption of late online corrections did not impair performance and may even have conferred a modest behavioral advantage. One possible interpretation is that, under normal conditions, participants occasionally generated late corrective movements that overshot the required adjustment, thereby introducing additional motor variability. Briefly preventing these corrections may therefore have promoted earlier commitment to the planned interception trajectory, reducing variability rather than increasing it. Although this effect was restricted to the shortest freezing duration, it suggests that altering the availability of late online corrections can modify the balance between predictive planning and corrective control. This interpretation is consistent with two-component models of aimed movement, in which visually guided corrections improve performance only when their benefits outweigh the variability they introduce (Elliott et al., 2001; de Lussanet et al., 2004). Likewise, optimal feedback control predicts that reliance on late sensory corrections becomes progressively less advantageous as the time available to implement them decreases (Franklin and Wolpert, 2008; Liu and Todorov, 2007). Consistent with this view, the largest freezing durations produced the greatest increase in landing-referenced manual error, suggesting that predictive planning alone was insufficient to fully compensate for the loss of late corrective movements.

Beyond its effects on interception accuracy, cursor freezing systematically altered the temporal organization of manual control. Unlike delayed-feedback paradigms, in which the relationship between hand movement and its visual consequences remains available and can gradually be recalibrated (Knelange and López-Moliner, 2020), cursor freezing temporarily interrupted the mapping between hand movement and cursor motion, while visual information about the target remained continuously available. During the frozen interval, no corrective command could influence the cursor position. Participants responded by reorganizing behavior before the perturbation occurred. Manual error relative to the target’s future landing position consistently decreased before freeze onset. Rather than tracking the target’s location, participants committed earlier to where it would ultimately arrive. This pattern closely matches predictions from optimal feedback control: when late sensory feedback becomes less useful, the optimal policy shifts control toward feedforward planning by reducing the contribution of late corrective commands and increasing anticipatory commitment (Franklin and Wolpert, 2008; Liu and Todorov, 2007; Todorov and Jordan, 2002).

We detected behavioral reorganization within only a few trials. Rather than developing gradually over repeated trials, the shift toward earlier manual commitment was already detectable during the first trials of each freezing block, indicating that participants adopted a new control policy soon after the altered task dynamics were introduced. Analysis of mouse movements during the frozen interval supported the same conclusion. As experience accumulated within a block, participants progressively reduced movements that no longer influenced cursor position, showing that they rapidly learned not only when corrective commands became ineffective but also where it was no longer worthwhile to generate them. Predictive planning improved behavior under the new constraint but could not restore interception accuracy to baseline, emphasizing that anticipatory control and online correction make complementary rather than interchangeable contributions to successful interception. Unlike previous studies, which inferred greater reliance on prediction primarily from endpoint performance under delayed or perturbed feedback (Galea et al., 2011; Knelange and López-Moliner, 2020; Shadmehr and Mussa-Ivaldi, 1994), the present approach quantified directly how behavior shifted from continuous target tracking toward earlier commitment to the predicted landing position throughout the movement.

The oculomotor response was more difficult to interpret. Gaze exhibited weaker and later reorganization than manual behavior, but the present data do not justify concluding that predictive reweighting was smaller in the oculomotor system. In fact, the omnibus analyses provided no evidence that the magnitude of the freeze-dependent modulation differed between effectors. The present results therefore support a dissociation between manual and gaze behavior in their temporal profiles and the detectability of freezing-induced changes, rather than in the absolute magnitude of those changes. Several explanations remain plausible. Gaze may already operate under a highly predictive policy during interception, leaving relatively little scope for additional reweighting when late manual corrections become unavailable (Senot et al., 2008; Zhao et al., 2019). Alternatively, gaze may continue to prioritize the moving target because maintaining an accurate estimate of its trajectory remains beneficial despite reduced opportunities for manual correction. A third possibility, more methodological: predictive reweighting may indeed occur in the oculomotor system but remain obscured because gaze simultaneously serves several competing functions, including target tracking, prospective sampling of future interception locations, and monitoring of the evolving manual response. Averaging across trials inevitably combines these processes into a single trajectory, reducing sensitivity to any one component. Consequently, the present findings should be interpreted as evidence that predictive reorganization was more readily detectable in manual trajectories than in gaze, rather than evidence that the oculomotor system failed to adapt.

This interpretation is compatible with a hierarchical organization of visuomotor control. Our findings are consistent with the idea that visual representations remain centered on the target’s current position while motor planning progressively incorporates information about the future interception point. Within this framework, the posterior parietal cortex continues to represent ongoing target motion, whereas premotor and motor regions progressively represent the predicted future state required for successful interception (Andersen and Buneo, 2002; Senot et al., 2008). Constraining late motor correction would therefore require little modification of visual sampling itself because compensation occurs primarily within downstream manual motor planning circuits. We emphasize, however, that this interpretation remains hypothetical. The present study measured behavior rather than neural activity, and the proposed mapping onto parietal and premotor mechanisms should therefore be regarded as a potential mechanistic interpretation consistent with the behavioral data rather than a direct experimental demonstration.

A broader implication emerges from these findings. Predictive control is often viewed as an additional computational process layered onto an otherwise reactive control system (Franklin and Wolpert, 2011; Shadmehr et al., 2010; Wolpert et al., 1995; Wolpert and Flanagan, 2001). Our results instead support a complementary perspective: prediction may arise naturally from the unavoidable delays that separate sensory input from motor output. Neural transmission, sensorimotor processing, and movement execution together introduce delays of tens to hundreds of milliseconds (Kawato, 1999; Todorov and Jordan, 2002; Wolpert et al., 1995), making purely reactive interception physically impossible. Cursor freezing extends the interval over which corrective commands become ineffective, and the motor system responds by committing behavior earlier. In this sense, predictive control is not an optional strategy recruited only under unusual circumstances, but a continuous adaptation to the temporal limitations of biological control (Márquez and Treviño, 2026). Cursor freezing, therefore, did not create predictive behavior. It shifted the weighting of a policy that is always present, thereby making it measurable. This interpretation extends forward-model accounts of interception (de Lussanet et al., 2004), and provides a behavioral framework for directly measuring the dynamic redistribution of predictive and feedback-based control.

Several limitations should be considered. First, we did not determine whether the behavioral reorganization induced by cursor freezing persisted after normal visuomotor coupling was restored. Consequently, our design could distinguish the control policy adopted during cursor freezing from baseline behavior, but it could not determine whether prolonged exposure produced lasting changes that persisted beyond the manipulation. Second, the target motion followed a highly regular gravity-like trajectory. This provided tight experimental control over predictive demands but reduced ecological complexity. More variable or less predictable target dynamics may reduce the extent to which participants can shift control toward earlier prediction (Márquez et al., 2024). Moreover, the eye-tracking analyses are subject to additional constraints. Gaze was analyzed at the level of continuous trajectories. We did not classify individual oculomotor events such as microsaccades, catch-up saccades, or brief predictive fixations directed toward the future interception location. Likewise, the landing-referenced and target-referenced coordinate systems necessarily converge near the end of the trial, making the late portion of the landing-referenced error trajectory unsuitable for between-condition comparisons. For this reason, all inferential analyses focused on the pre-freeze interval, where target-centered (RMSE_CC_/RMSE_GC_) and landing-centered (RMSE_CL_/RMSE_GL_) reference frames remained functionally distinct.

The task was also intentionally designed to investigate the spatial component of predictive interception. Because fall duration was fixed across trials, participants did not need to estimate time-to-contact, a variable that many interception studies consider fundamental and often derived from optical information and priors on gravitational forces (Gómez and López-Moliner, 2013; Zago et al., 2009). Consequently, the present findings should be interpreted as evidence for changes in the spatial organization of predictive control rather than as a general account of temporal interception. Finally, previous work has shown substantial individual differences in the relative weighting of predictive planning and online correction during this task (Arthur et al., 2024; Márquez et al., 2024). The present analyses emphasize population-level behavior and therefore did not determine whether the magnitude of the observed redistribution depends on each participant’s baseline control strategy.

Beyond the specific findings of this study, the methodological approach provides a general framework for investigating predictive control. By combining cursor freezing with continuous tracking-error analyses referenced to both the target’s instantaneous position and its predicted landing position, and validating these measures with a one-parameter mixture-control simulation, we dissociated predictive and feedback-dependent components of ongoing behavior without altering the visual information provided by the target. Simultaneously evaluating error relative to both the target’s instantaneous position and its future landing position allowed us to characterize not only how accurately participants tracked the target, but also when behavior became committed to its predicted future state. This temporal perspective provides a direct behavioral measure of how behavior shifts from online tracking toward predictive commitment when feedback contingencies change. This framework may also have practical relevance beyond laboratory interception tasks. Many real-world human–machine interactions, including brain-computer interfaces, teleoperation, robotic assistance, and advanced prosthetic control, involve delayed or intermittently ineffective motor commands (Knelange and López-Moliner, 2020; Straube et al., 2017). Under these conditions, successful performance increasingly depends on predictive planning rather than continuous correction. Our results identify a measurable behavioral signature of this transition, an earlier reduction in error relative to the predicted endpoint, that may provide an objective indicator of when users shift from feedback-dominated control toward anticipatory planning. Whether this measure can ultimately serve as an adaptive control signal remains an important question for future work.

To conclude, these findings show that transiently disrupting the effectiveness of late motor correction does not eliminate predictive control but instead shifts behavior toward earlier commitment to the predicted landing position when online correction becomes unreliable. The resulting behavioral shift is rapid, incomplete, and expressed most clearly in manual trajectories, where earlier commitment to the future interception point becomes directly observable. More broadly, the present work suggests that predictive control is best understood not as a separate mode of action, but as the dynamic reallocation of control toward future states whenever biological (or technological) constraints render immediate sensory feedback less useful. Cursor freezing, therefore, provides a simple experimental manipulation for studying how behavior shifts between predictive planning and online correction during a movement.

## Data Availability

The datasets presented in this study can be found in online repositories. The names of the repository/repositories and accession number(s) can be found at: https://osf.io/t42hc/?view_only=811b571a2ce943eeb3ae078ed77b2568.
