# Supplementary material for: Preventing late corrections reveals anticipatory motor control
Source: Front Syst Neurosci. 2026 Aug 28;20:1892135. doi: 10.3389/fnsys.2026.1892135 (PMC13561802; doi:10.3389/fnsys.2026.1892135)
Supplement: Supplementary file 1 [file Data_Sheet_1.PDF]

Supplemental Material

for

Preventing Late Corrections Reveals Anticipatory Motor Control

Running title: Cursor-Freezing Enhances Prediction

Inmaculada Márquez[1,2,4], Luis Lemus[3*], Mario Treviño[4*]

[1]Departamento de Psicología, Centro Universitario de la Ciénega, Universidad de Guadalajara, Ocotlán, México
[2]Laboratorio de Neurofisiología, Departamento de Bioingeniería Traslacional, Centro Universitario de Ciencias Exactas e Ingenierías, Guadalajara, Jalisco, México
[3]Departamento de Neurociencia Cognitiva, Instituto de Fisiología Celular, Universidad Nacional Autónoma de México, 04510, Ciudad de México, México
[4]Laboratorio de Plasticidad Cortical y Aprendizaje Perceptual, Instituto de Neurociencias, Universidad de Guadalajara, Guadalajara, Jalisco, México

KEYWORDS: Predictive visuomotor control; preemptive motor planning; target interception; gaze-hand coordination; sensorimotor prediction.

Correspondence:
*Dr. Luis Lemus (lemus@ifc.unam.mx), Departamento de Neurociencia Cognitiva, Instituto de Fisiología Celular, Universidad Nacional Autónoma de México, 04510, Ciudad de México, México; Dr. Mario Treviño (mario.trevino@academicos.udg.mx), Laboratorio de Plasticidad Cortical y Aprendizaje Perceptual, Instituto de Neurociencias, Universidad de Guadalajara. Francisco de Quevedo 180, Arcos Vallarta. 44130. Guadalajara, Jalisco, México.

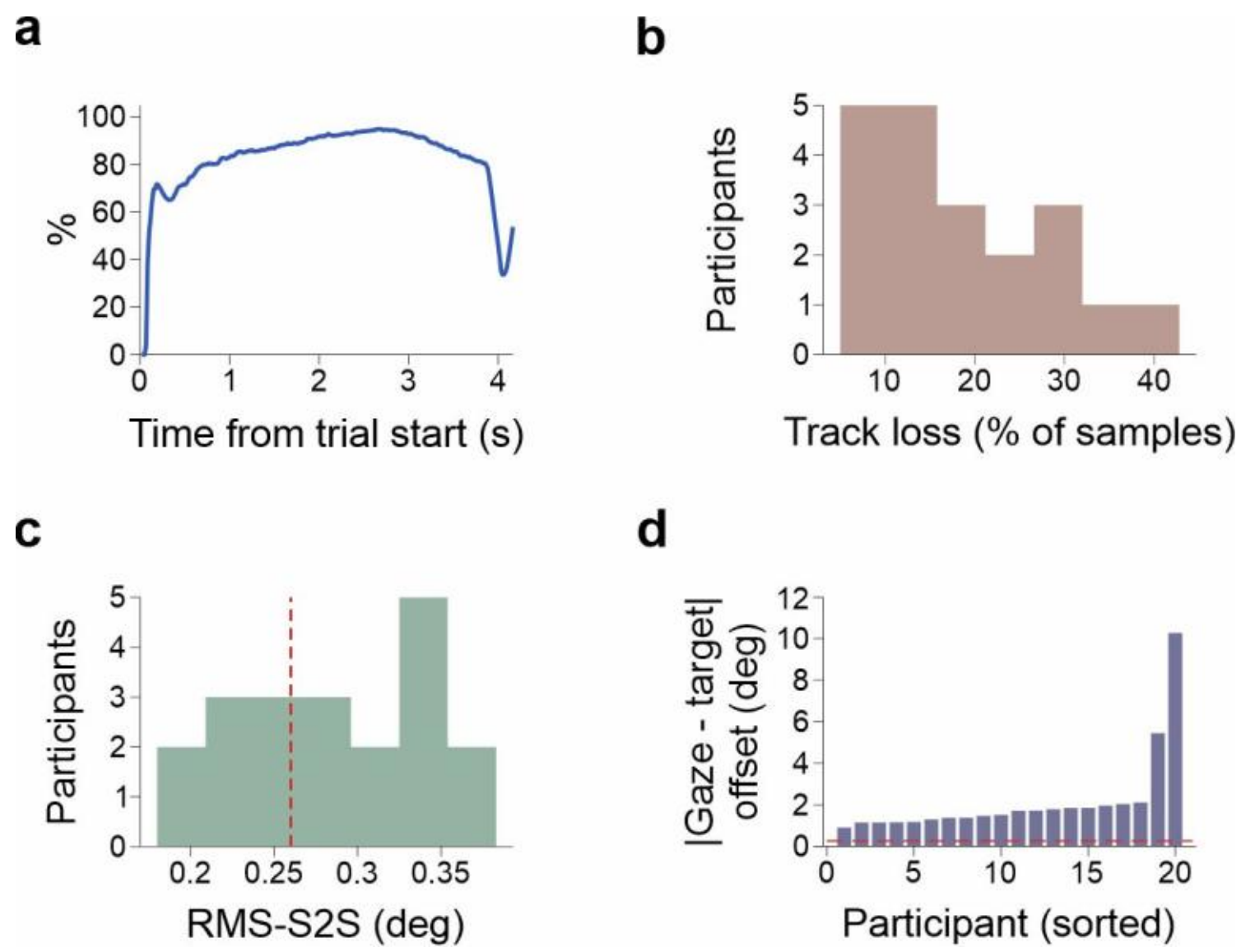

Supplementary Figure 1. Eye-tracking data quality. Achieved quality for gaze recordings (60-Hz remote tracker; 33.80 px/deg at screen center, 36.40 px/deg averaged across the display; nominal spec resolution 0.26°; target size 0.3°). (**a**) Per-participant track loss (mean 18.4%, median 16.6%, range 5.7–42.4%). (**b**) Per-participant sample-to-sample precision (RMS of sample-to-sample deviation over 300-ms windows; median 0.286°, range 0.192–0.377°); the dashed line marks the 0.26° spec, exceeded by 12 of 20 participants. (**c**) Per-participant accuracy proxy, median horizontal gaze–target offset during the initial fixation epoch (systematic offset median +0.48°, range −5.40 to +1.33°; absolute value, an upper bound, median 1.61°). (**d**) Sample validity across the trial: complete-case coverage is lost only in the first six frames, so the usable window is frames 6–250 (0.10–4.17 s). Precision and accuracy were defined following (Holmqvist et al. 2011; Niehorster et al. 2020). The accuracy proxy is an upper bound on measurement error, since it also contains genuine oculomotor variability, and remote trackers with unrestrained participants degrade in these terms (Niehorster et al. 2018).

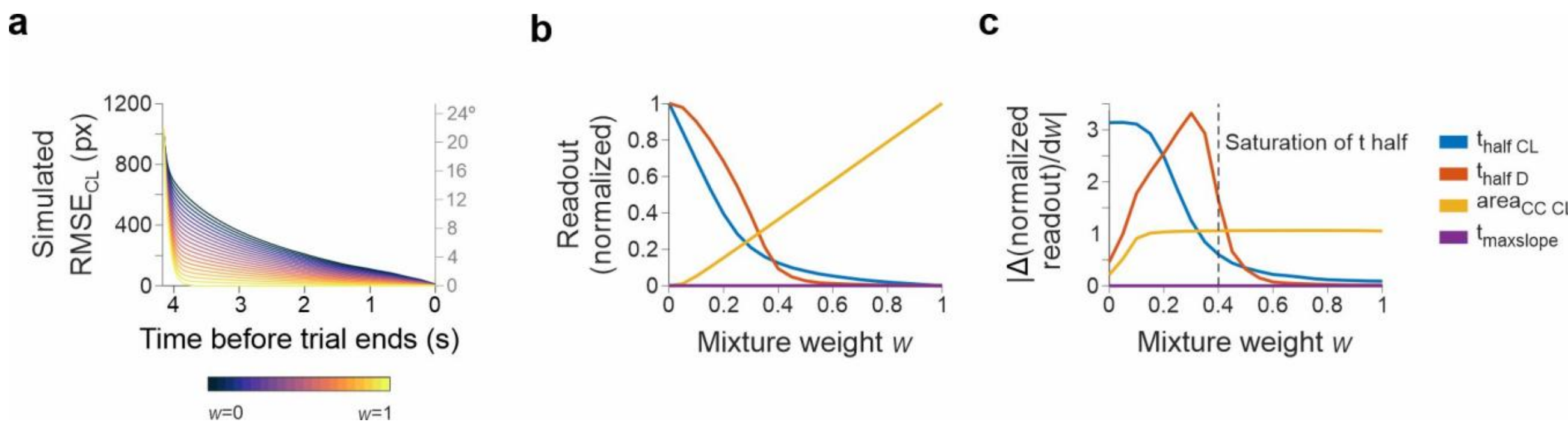

Supplementary Figure 2. Local sensitivity of the mixture-controller read-outs. Sensitivity of the RMSE read-outs to the underlying control weight, evaluated on a fine grid (w = 0:0.05:1; 21 weights; 500 synthetic trials per weight driven by the real target trajectories; first-order plant $\tau = 5$ frames, 83.3 ms; read-out window, frames 1–200). The controller time constant was fixed at $\tau = 83.3$ ms, corresponding to five display frames, rather than fitted. This value is below the latency of visually guided manual correction, which begins roughly 100–150 ms after a target displacement (Oostwoud Wijdenes et al. 2011), and was chosen so that the simulated controller is not slower than the system it stands in for. Because τ is fixed rather than estimated, the simulation serves as a calibration device, not a model fit. We verified that the ordering of conditions along the recovered weight axis is unchanged for τ between 50 and 150 ms. (**a**) Half-fall time of $RMSE_{CL}$ and of the difference trace $\Delta = RMSE_{CL} - RMSE_{CC}$ as a function of w. (**b**) Local sensitivity |d(read-out)/dw|, each normalized to its own maximum; the dashed line marks the 20%-of-maximum saturation threshold. The half-fall time of RMSECL drops below threshold beyond $w \approx 0.40$ (usable range 0.00–0.35), and the difference trace beyond $w \approx 0.45$, whereas the area between the $RMSE_{CC}$ and $RMSE_{CL}$ curves never saturates. Monotonicity: Spearman $r = -1.00$ for both half-fall read-outs and +1.00 for the area; flatness CV = 1.16, 1.22, and 0.22, respectively. (**c**) Area between the $RMSE_{CC}$ and $RMSE_{CL}$ curves versus w, the non-saturating corroborating read-out. (**d**) Per-subject inverted weight $\hat{w}$ for each condition (control, n = 60; each freeze duration 10–50 frames, n = 30; see Methods for the composition of the two samples), obtained by inverting the simulated half-fall(w) mapping; the shaded band marks the saturating region ($w > 0.40$). All conditions fall at $\hat{w}$ = 0.00–0.01, within the steep part of the curve. Paired shifts (freeze − control): 10 frames $t_{(29)} = 1.27$, $p = 0.215$, $d = 0.23$; 20 frames $t_{(29)} = 2.27$, $p = 0.031$, $d = 0.41$; 30 frames $t_{(29)} = 2.64$, $p = 0.013$, $d = 0.48$; 40 frames $t_{(29)} = 2.86$, $p = 0.008$, $d = 0.52$; 50 frames $t_{(29)} = 2.54$, $p = 0.017$, $d = 0.46$. Data are mean ± SEM; tests two-tailed.

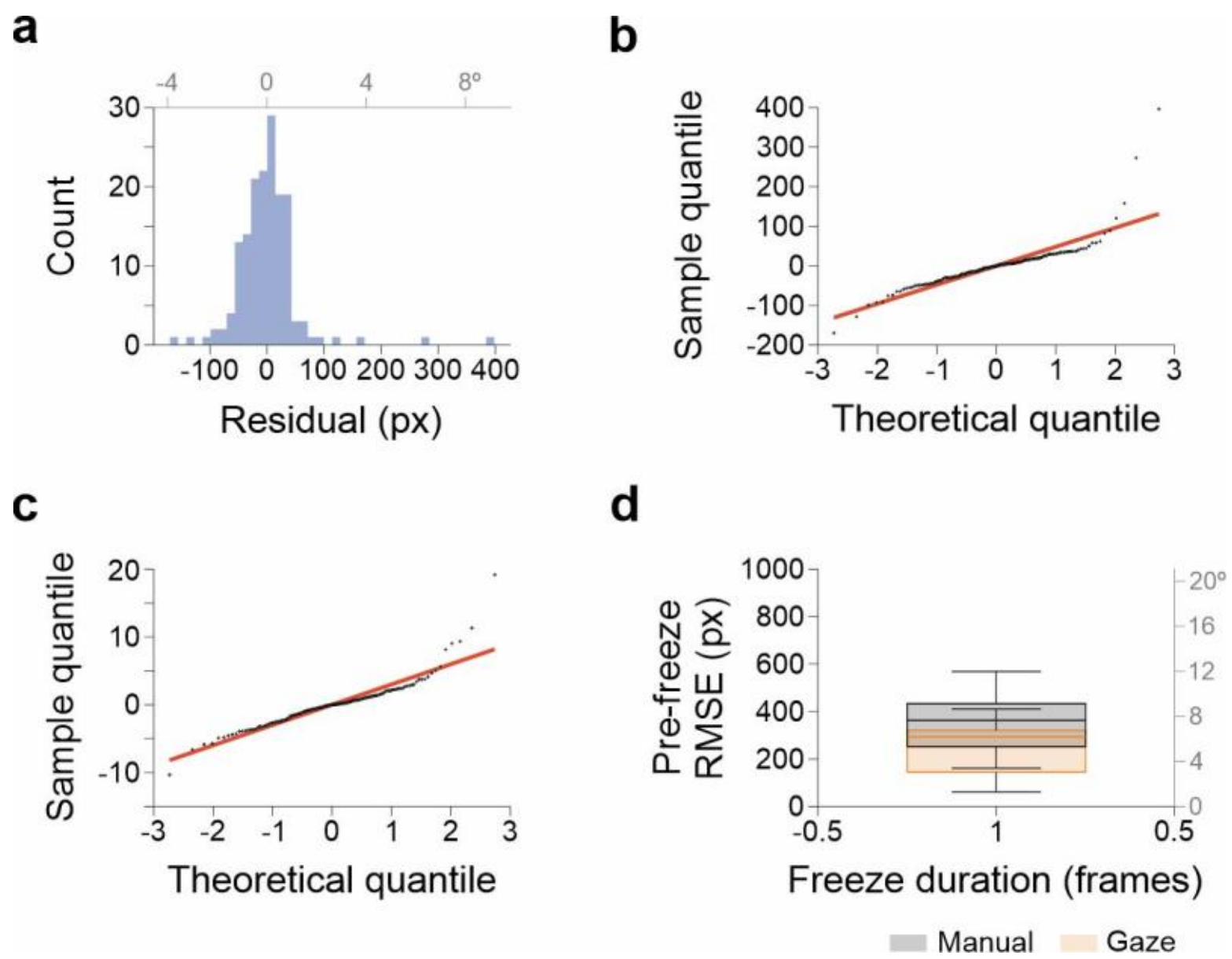

Supplementary Figure 3. Omnibus linear mixed-effects model on pre-freeze RMSE. Omnibus model RMSE ~ Condition × Epoch × Effector × RefFrame + (1 | subject), fit by REML with Satterthwaite degrees of freedom on the cohort carrying simultaneous cursor and gaze recordings. This is the four-way model referred to in the main text; the three-way models quoted there (df ≈ 840) were fitted on the full sample and are not shown here. (**a**) Distribution of residuals from the omnibus linear mixed-effects model of pre-freeze RMSE (RMSE ~ Condition × Epoch × Effector × RefFrame + (1|subject), REML), in pixels. (**b**) Forest plot of the estimable fixed effects: Effector $F_{(1,\ 133)} = 166.6$, $p < 0.001$; RefFrame $F_{(1,\ 133)} = 16.8$, $p < 0.001$; Epoch $F_{(1,\ 133)} = 5.68$, $p = 0.019$; Effector × RefFrame $F_{(1,\ 133)} = 57.0$, $p < 0.001$; the remaining two- and three-way terms are non-significant ($p \geq 0.57$). (**c**) Model comparison: the omnibus versus a model without the four-way term (likelihood ratio LR = 80.0, Δdf = 2, $p < 0.001$) and versus the reduced fixed-effects structure reported in the main text (no term changes significance); adding a by-effector random slope improves fit (AIC 1624.1 vs 1640.8). (**d**) Residual diagnostics (skewness +1.01, kurtosis 5.81; Box–Cox $\lambda = 1.16$); the inferential pattern holds under the variance-stabilizing transform.

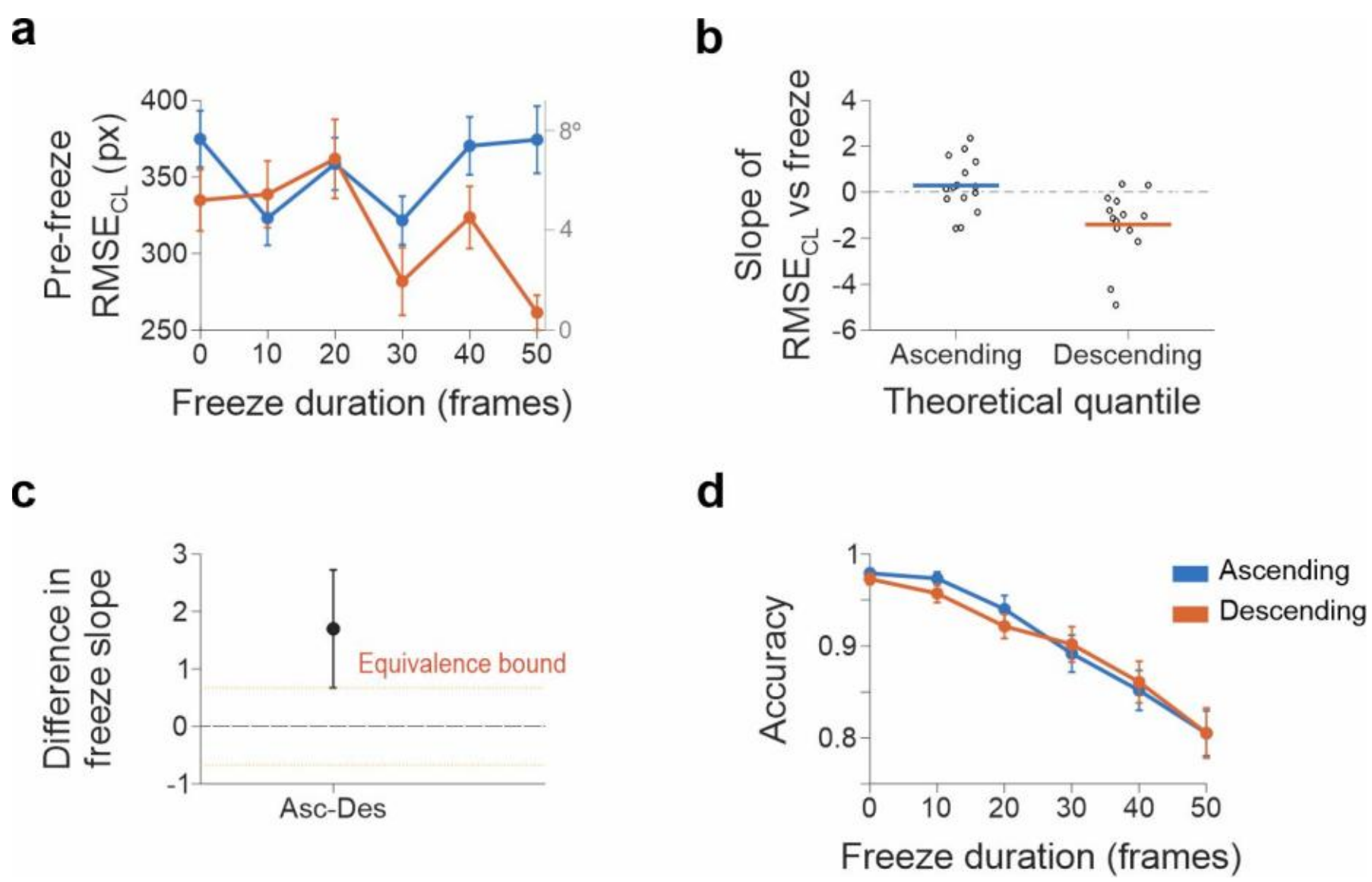

Supplementary Figure 4. Effect of block order (ascending *vs* descending freeze sequence). Block-order analysis on the blocked cursor sessions, head blocks only. (**a**) Interception accuracy versus freeze duration for the ascending and descending sequences. Order has no main effect (LME $F_{(1,80.9)} = 0.005$, $p = 0.94$; trial-level GLMM $F_{(1,\,11592)} = 0.05$, $p = 0.82$) and no Order × Freeze interaction on accuracy ($F_{(1,319)} = 1.00$, $p = 0.32$), but a three-way Epoch × Order × Freeze term is present ($F_{(1,319)} = 5.04$, $p = 0.025$; GLMM $F = 9.74$, $p = 0.002$). (**b**) Pre-freeze RMSE_{CL} versus freeze duration for the two sequences. Order has no main effect ($F_{(1,49.3)} = 0.007$, $p = 0.94$), but Order × Freeze is significant ($F_{(1,319)} = 6.31$, $p = 0.013$): freeze sensitivity differs between directions. (**c**) Per-participant slope of pre-freeze RMSE_{CL} on freeze duration, ascending versus descending (ascending +0.29 ± 1.16, $t_{(14)} = 0.98$, $p = 0.35$; descending −1.41 ± 1.52, $t_{(13)} = -3.46$, $p = 0.004$; group difference +1.70, 95% CI [0.67, 2.73], $t_{(27)} = 3.40$, $p = 0.002$, Hedges $g = 1.23$; two one-sided tests do not establish equivalence, $p = 0.98$). Under both sequences, the biphasic pre-freeze RMSE_{CL} pattern relative to control was present; the sequences differed in how steeply that pattern scaled with freeze duration, not in whether it occurred. (**d**) Fixed-effect coefficient tables for the accuracy and RMSE_{CL} models. Data are mean ± SEM; tests two-tailed.

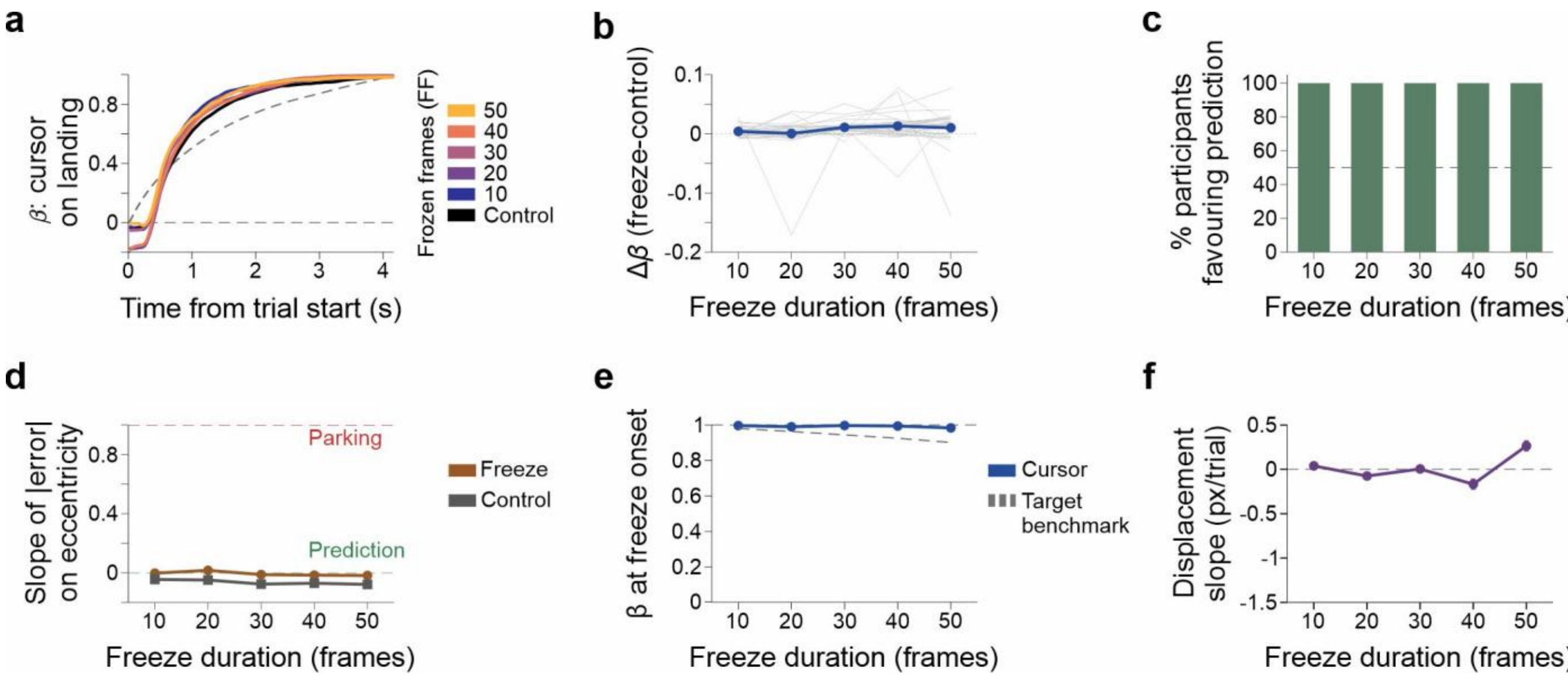

Supplementary Figure 5. Trial-specific prediction versus a fixed-parking strategy, and within-block decline of movement during the freeze. Regression of the cursor position at freeze onset on the landing position of the same trial (slope β = 1, full trial-specific prediction; β = 0, parking at a fixed location), late-block trials, $n = 60$ participants; each freeze condition is compared with control at the identical pre-freeze frame, so the contrast is not confounded with time in the trial. (**a**) β for each freeze condition and for control at the matched frame, with the target's own slope on landing overlaid as a benchmark. β is high throughout (0.98–1.00) and exceeds the target benchmark at every duration (30 frames +0.053, $t_{(59)} = 31.1$, $p < 0.001$, $d = 5.67$; 50 frames +0.082, $t_{(59)} = 13.7$, $p < 0.001$, $d = 2.50$), so the cursor carries more trial-specific landing information than the display itself, a pattern neither tracking nor parking can produce. (**b**) Difference in β between freeze and control at the matched frame: positive, i.e. the cursor follows the trial-specific landing *more*, not less, when a freeze is coming (30 frames $t_{(59)} = 5.16$, $p < 0.001$, $d = 0.94$; 40 frames $t_{(59)} = 2.80$, $p = 0.009$, $d = 0.51$). (**c**) Per-participant model comparison, parking (intercept only) versus trial-specific prediction: prediction is favored in 100% of participants at every freeze duration (median ΔBIC +124.7 to +156.4), and the parking null (β = 0) is rejected in 100%. (**d**) Eccentricity index — slope of interception error on landing eccentricity (1 = parking, 0 = prediction): near zero under freezing (−0.017 to +0.017) and below control at every duration, far from the value of 1 that fixed parking necessarily produces on extreme lateral landings. (**e**) Commitment time — the moment the cursor's slope on landing overtakes the target's own, relative to control: earlier under freezing at the shortest and longest durations (10 frames −0.37 s, $t_{(59)} = -3.51$, $p = 0.002$, $d = -0.64$; 50 frames −0.32 s, $t_{(59)} = -3.72$, $p < 0.001$, $d = -0.68$). Data are mean ± SEM; tests two-tailed. (**f**) Within-block decline in cursor displacement during the freeze. For each freeze duration, the integrated absolute frame-to-frame displacement over the freeze was regressed on the trial's serial position within its block with a linear mixed model including by-participant random intercepts. Slopes were negative at every freeze duration, indicating that participants progressively withheld movements that could no longer influence the cursor.

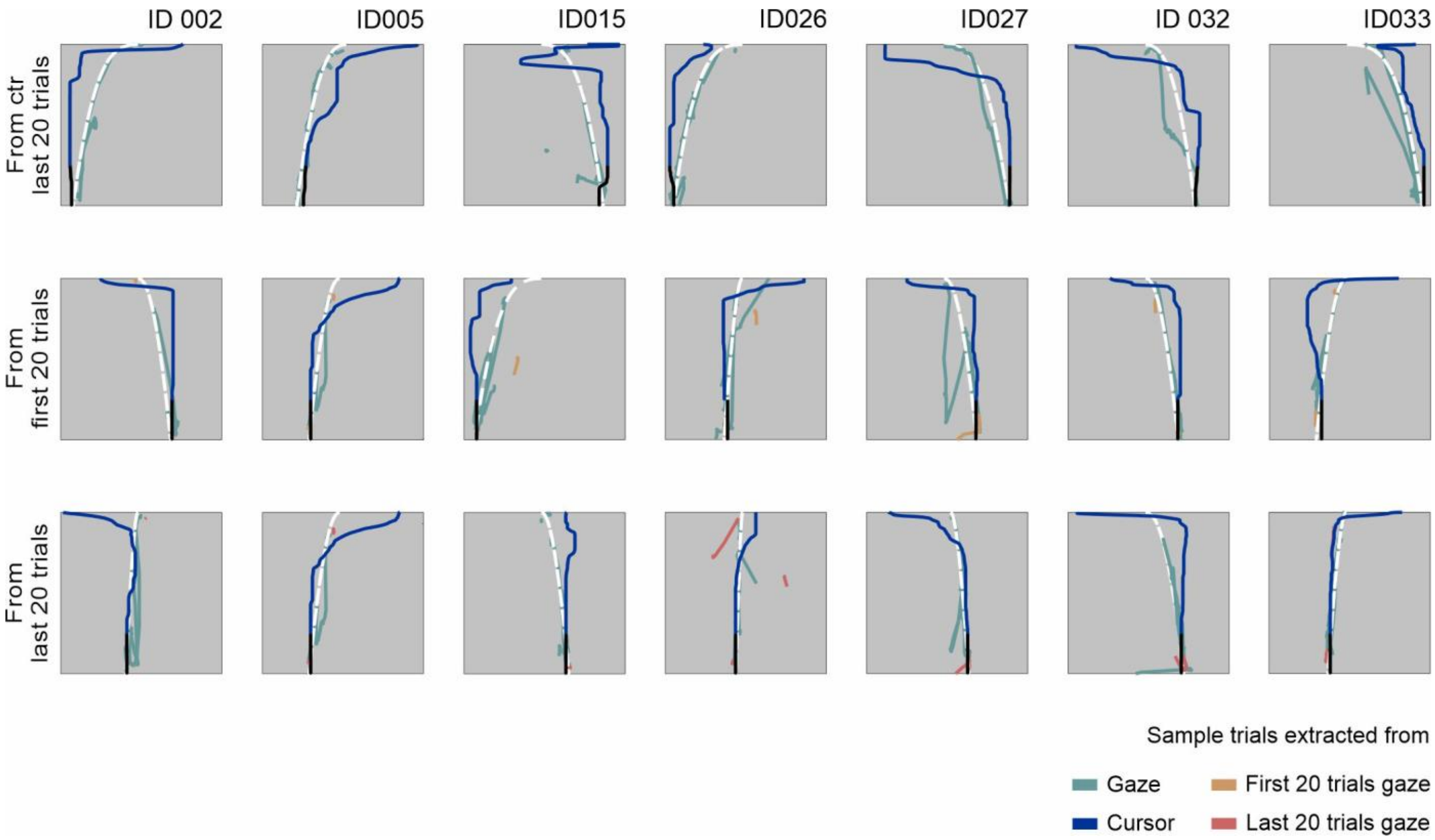

Supplementary Figure 6. Individual cursor trajectories under cursor-freezing. Cursor trajectories from seven representative participants (one per column) in the control condition (upper row), early freezing trials (middle row), and late freezing trials (lower row), at the longest freeze duration (50 frames, 833 ms). Dashed lines, target trajectories; vertical markers, freeze onset (in black). Participants were selected to span the range of pre-freeze $RMSE_{CL}$ slopes and are not a random subsample.

Supplemental Bibliography

Holmqvist K, **Nyström M**, **Andersson R**, **Dewhurst R**, **Jarodzka H**, **Weijer J van de**. *Eye Tracking: A comprehensive guide to methods and measures*. OUP Oxford, 2011.

Niehorster DC, **Cornelissen THW**, **Holmqvist K**, **Hooge ITC**, **Hessels RS**. What to expect from your remote eye-tracker when participants are unrestrained. *Behav Res Methods* 50: 213–227, 2018.

Niehorster DC, **Hessels RS**, **Benjamins JS**. GlassesViewer: Open-source software for viewing and analyzing data from the Tobii Pro Glasses 2 eye tracker. *Behav Res* 52: 1244–1253, 2020.

Oostwoud Wijdenes L, **Brenner E**, **Smeets JBJ**. Fast and fine-tuned corrections when the target of a hand movement is displaced. *Experimental Brain Research* 214: 453–462, 2011.
